# Comprehensive immune profiling of human peripheral blood mononuclear cells using two complementary spectral flow cytometry panels encompassing 60 unique markers

**DOI:** 10.3389/fimmu.2026.1894779

**Published:** 2026-09-01

**Authors:** Maartje H. Rietdijk, Leo C. Kuhnen, Marlous van den Braber, Alberta G. A. Paul, M. Fernanda Pascutti, Juan J. García-Vallejo

**Affiliations:** 1 Amsterdam UMC, Vrije Universiteit Amsterdam, Department of Molecular Cell Biology and Immunology, Cancer Center Amsterdam, Amsterdam institute for Immunology and Infectious diseases, Amsterdam, Netherlands; 2 Scientific Commercialization, Cytek Biosciences, Inc., Fremont, CA, United States; 3 Amsterdam UMC Immunotherapy Center, Amsterdam, Netherlands

**Keywords:** high dimensional data analysis, human, immunomonitoring, PBMC, spectral flow cytometer

## Abstract

Comprehensive immune profiling is essential for immunomonitoring studies aimed at identification of diagnostic and prognostic biomarkers. Peripheral blood mononuclear cells (PBMCs) undergo phenotypic and functional changes during disease, making them invaluable for the characterization of immune cell composition in both cross-sectional and longitudinal immune monitoring studies. We have developed a comprehensive immunophenotyping method based on two complementary panels of 60 unique markers to characterize B cells, T cells, innate lymphoid cells (ILCs), γδ T cells, mucosal-associated invariant T (MAIT) cells, natural killer (NK) cells, monocytes, dendritic cells (DCs) and several of their subsets, including their functional status, within human peripheral blood mononuclear cells (PBMCs). Dividing the markers over two complementary panels allows for inclusion of many more markers than currently allowed for single panels by state-of-the-art spectral flow cytometers, enabling the immunophenotyping of a broad spectrum of immune cell subsets at great analytical depth. This includes rare subsets and subsets that require an extensive combination of markers to be resolved, combined with the option of assessing differentiation, activation and exhaustion. The method enables the resolution of more than 50 distinct populations, and detailed exploration of differentiation, activation and exhaustion of these subsets, as is demonstrated here on PBMC samples from healthy donors, glioblastoma, inflammatory bowel disease and COVID-19 patients. The protocol supports both manual and unsupervised data analysis approaches and is suitable for large-scale immunomonitoring studies requiring standardized, reproducible multi-batch workflows.

## 1 Introduction

Peripheral blood mononuclear cells (PBMCs) comprise a diverse array of immune cell subsets that continuously circulate through the body, gathering information as they interact with cells in various tissues. In response to infections or disease, PBMCs undergo phenotypic and functional changes that reflect the health status and disease progression at the tissue level (1–3). This makes them valuable cells for studying changes in immune cell composition and function related to disease in both cross-sectional and longitudinal studies. Comprehensive immune profiling of PBMCs is essential for immune monitoring studies aiming to identify cellular diagnostic or prognostic biomarkers.

PBMCs are isolated from whole blood, which can be easily acquired with minimal discomfort for patients, especially when compared to tissue biopsies. Whole blood is considered the most complete biological fluid to study immune involvement in disease pathophysiology, as it contains a broad range of immune cells. However, neutrophils account for up to 60–65% of total white blood cells (4), posing a challenge for the analysis of rare immune cell subsets with potentially important immunoregulatory functions or cells migrating between tissues. Additionally, neutrophils do not survive cryopreservation, which complicates the logistics of longitudinal studies. While whole blood fixation after erythrocyte lysis may overcome batch effects and include all immune cells present in blood, fixation restricts the selection of antibodies to those that are fixation-resistant. Focusing on PBMCs enables the study of rare subsets and allows for cryopreservation rather than fixation, facilitating multi-batch studies and enabling functional assays.

Spectral flow cytometry is a powerful technique that measures the entire emission spectrum of fluorochromes across all laser lines and detectors available on a spectral flow cytometer. This wealth of information allows for the distinction of spectral signatures even when their peak channels are very similar. As a result, comprehensive immunophenotyping panels can be designed to cover a broad range of lineage, differentiation, and functional markers, enabling the resolution of numerous immune populations within a single sample. To date, antibody panels containing up to 45 (5) and 50 (6) markers have been described. Here, we aimed to develop an immune monitoring strategy based on spectral flow cytometry to identify as many known immune cell subsets as possible, including their activation and differentiation status. We designed two complementary panels that collectively include 60 unique markers for B cells, T cells, monocytes, dendritic cells (DCs), natural killer (NK) cells, mucosal-associated invariant T (MAIT) cells, γδ T cells, and innate lymphoid cells (ILCs), and several of their subtypes. By dividing the markers between two complementary panels, we include a greater total number of markers while still assessing the expression of differentiation markers across multiple lineages. This approach enables the immunophenotyping of a broad spectrum of immune cell subsets, including rare populations and those requiring extensive marker combinations for resolution, as well as the assessment of differentiation, activation and exhaustion states. Sample availability is not a limiting factor for the application of these panels, as one million PBMCs is sufficient for both panels, and a typical 5 mL blood draw yields approximately 2.5–10 million PBMCs. The panel presented here expand on previously published methods by combining phenotyping and functional markers to enable comprehensive assessment of more than 50 phenotypically unique and well-characterized cell populations and their activation and differentiation status from a single PBMC sample.

The protocol presented here is optimized for the Cytek Aurora 5-laser spectral flow cytometer and describes the full workflow from panel design through PBMC cryopreservation, staining, data acquisition, quality control, batch normalization, and both manual and unsupervised data analysis. Applications of this methods on comparable spectral instruments, such as the ID7000 (Sony Biotechnology), Attune Xenith (ThermoFisher Scientific), NovoCyte Opteon (Agilent), FACSymphony (Becton, Dickinson (DB) Biosciences), or CytoFLEX (Beckman Coulter), is possible but in that case additional optimization of the protocols is strongly advised.

## 2 Materials

### 2.1 Biological samples

Peripheral Blood Mononuclear Cells (PBMCs) were isolated by density gradient centrifugation using Lymphoprep™ (Axis-Shield, United Kingdom) from buffy coats of healthy volunteers (n = 8) purchased at Sanquin (Amsterdam, the Netherlands), according to reagent manufacturer’s instructions. In short, buffy coats were diluted in an equal volume of 1% citrate in PBS. 30 ml of diluted blood was carefully pipetted over 15 ml of Lymphoprep in sterile 50 ml conical tubes and centrifuged in a swing-out rotor for 30 minutes at 800 x *g* and 22 °C with deceleration set to 1. PBMCs were collected with a Pasteur pipette from the sample/medium interface and transferred to a sterile conical tube. Additionally, whole blood samples were collected in 10 ml cellular preparation tubes (CPT) (BD Biosciences, Plymouth, UK) from glioblastoma (GBM) (n = 7), chronic inflammation (CI) (n = 6), and COVID-19 patients (n = 6). PBMCs were isolated by density gradient centrifugation as described elsewhere (7). In short, CPTs were centrifuged at 1800 x *g* for 20 minutes at room temperature. Then, tubes were gently inverted to mix PBMC and plasma residing above the gel plug and transferred to a sterile 50 ml conical tube. PBMCs were washed twice with 1% citrate in PBS (Fresenius Kabi, Huis ter Heide, The Netherlands), and resuspended in freezing medium, consisting of 10% DMSO (Sigma-Aldrich, Darmstadt, Germany) in FBS (Serana, Brandenburg, Germany). Aliquots of cells were stored in cryotubes (10-50*10^6 cells/vial) in -80 °C and were then moved to LN_2_ until analysis.

### 2.2 Reagents and materials for staining protocol

- 96-well V-bottom polypropylene microplate (Greiner Bio-One, catalogue # 651201) or equivalent.
- 10, 200 and 1000 µl pipet tips.
- 10, 20, 200 and 1000 µl pipettors.
- 1.5 ml eppendorf tube (ThermoFisher Scientific, catalogue #3451PK) or equivalent.
- RPMI 1640 (Gibco^®^, ThermoFisher Scientific, catalogue #11875093).
- Foetal Bovine Serum (FBS) (Serana, calatog #S-FBS-CO-015).
- Phosphate Buffered Saline (PBS) (Fresenius Kabi).
- Bovine Serum Albumin (BSA) (Sigma-Aldrich, catalogue #A9418-500G) or equivalent.
- BD Horizon™ Brilliant Stain Buffer (BD Biosciences, catalogue #566349).
- True-Stain Monocyte Blocker™ (BioLegend, catalogue #426103).
- BD Pharmingen™ Human Fc Block™ (BD Biosciences, catalogue #564220).
- UltraComp eBeads™ Compensation Beads (ThermoFisher Scientific, catalogue #01-2222-41).
- eBioscience™ Foxp3/Transcription Factor Staining Buffer Set (ThermoFisher Scientific, catalogue #00-5523-00).
- Thawing medium (RPMI 1640 supplemented with 10% FBS).
- Wash/stain buffer (PBS with 0.05% Bovine Serum Albumin).
- Vendor and catalogue numbers of antibody and additional reagents used in the panels are provided in **Table 2**.

**Table 1:** Marker combinations for target populations and functional status of the subsets for the panels described in this study.

Antibodies	Purpose
Panel 1	
CD3	Pan T cell marker
TCRγδ, TCRVδ2, CD3	γδ1/3 and γδ2 T cells
CD3, TCRVα7.2, TCRVα24J18	iNKT
CD3, TCRVα7.2, CD8, CD161	MAIT
CD3, iNKT, TCRVα7.2, TCRγδ, TCRVδ2, CD56	NKT-like
CD3, CD8	Cytotoxic T cells
CD3, CD4	Helper T cells
CD3, CD8, CD4, CD45RA, CCR7, CD95	Naive T cells
CD3, CD4, CD8, CD45RA, CCR7, CD27, CD28	Central memory
CD3, CD4, CD8, CD45RA, CCR7	TEMRA and effector memory
CD3, CD4, CD8, CD45RA, CCR7, CD27, CD95	SCM
CD3, CD4, CD8, CD45RA, CCR7, CD27, CD28	Early like, early effector, intermediate effector and terminal effector
CD3, CD4, CXCR5, FoxP3, CD25	Tregs
CD3, CD4, CXCR5, CD25	TFH
CD3, CD8, CXCR5	CXRC5^+^ CD8 T
CD56, CD16	Early, mature and terminal NK cells
CD56, CD16, CD127, CRTH2, cKIT	ILC1 ILC2 ILC3
CD69	T cell and NK cell activation
CD39	Tregs Aativation and effector function
TIGIT, PD-1, CTLA-4, TIM-3	T cell exhaustion
LAG3	T cell exhaustion, NK eeffector function
KI67	Proliferiation potential
CD57	NK cell maturation
NKG2a, NKG2c	NK cell differentiation
NKp44	NK cell, ILC and NKT-like cell activation
Panel 2	
CD19, CD20	B cells
CD19, CD20, IgD, CD10, CD24	Transitional B cells
CD19, CD20, IgD, IgM	Marginal zone B cells
CD19, CD20, IgD, CD27, CD24, CD38	Naive B cell
CD19, CD20, IgD, CD24, CD38, PD-L1	IgD^+^CD24loCD38^+^/^-^B/regulatory B cells
CD19, CD20, IgD, CD38	Plasmablasts
CD19, CD20, IgD, CD27	Memory B cells
CD19, CD20, IgD, IgG, IgM, CD24, CD38	Memory and activated IgM^+^ B cells, IgG^+^ B cells, and IgM^-^IgG^-^ B cells
CD19, CD11c, HLA-DR, CD123	Basophils
CD19, CD11c, CD141	cDC1, cDC2
CD19, CD11c, CD303	pDC
CD19, CD11c, CD14, CD16	Classical, intermediate and non-classical monocytes
CD19, CD14, HLA-DR, CD11b, CD33	eMDSC
CD40, CD24, CD73, FcRL4, FcRL5	B cell activation and differentiation
CD80, CD86, CD169, CD163	Monocyte activation

**Table 2:** Reagents used for panels presented in this study.

A					
Specificity	Fluorchrome	Clone	Vendor	Catalog number	Titer (ng/test)
CCR7	BV421	G043H7	BD Biosciences	747843	560
CD123	Superbright 436	6H6	ThermoFisher	62-1239-42	250
CD127	APC-R700	HIL-7R-M21	BD Biosciences	565185	200
CD14	Superbright 436	61D3	ThermoFisher	62-0149-42	250
CD16	BUV496	3G8	BD Biosciences	612944	250
CD161	eF450	HP-3G10	ThermoFisher	48-1619-42	500
CD19	Superbright 436	HIB19	ThermoFisher	62-0199-42	1000
CD1c	Superbright 436	L161	ThermoFisher	62-0015-42	500
CD25	PE-AlexaFluor700	CD25-3G10	ThermoFisher	MHCD2524	100
CD27	APC-H7	M-T271	BD Biosciences	560222	250
CD28	BV650	CD28.2	BioLegend	302946	200
CD3	Spark Blue 550	SK7	BioLegend	344852	400
CD39	BUV661	TU66	BD Biosciences	749967	500
CD4	cFluor YG584	SK3	Cytek	SKU R7-20041	10
CD45	PerCP	2D1	BioLegend	368506	200
CD45RA	BUV395	5H9	BD Biosciences	740315	50
CD56	Spark 685 NIR	5.1H11	BioLegend	362564	500
CD57	Fitc	HNK-1	BioLegend	359604	250
CD69	BUV737	FN50	BD Biosciences	612817	400
CD8	APC-Fire810	SK1	BioLegend	344764	250
CD95	PE-Cy5	DX2	BioLegend	305610	125
cKIT	BV605	104D2	BD Biosciences	562687	400
CRTH2	PE-Cy7	BM16	BioLegend	350118	1000
CTLA-4	Alexa Fluor 647	BNI3	BioLegend	369626	32
CXCR5	BV750	J252D4	BioLegend	356942	100
Foxp3	BB700	236A/E7	BD Biosciences	566526	1200
iNKT (TCR Vα24-Jα18)	cFluor BYG710	Va24-Ja18	Cytek	Custom*	250
Ki67	BV510	B56	BD Biosciences	563462	750
Lag3	BV711	11C3C65	BioLegend	369320	1000
NK2GA	APC	REA110	Miltenyi	130-113-563	300
NKG2c	PE	REA205	Miltenyi	130-119-776	200
NKp44 (CD336)	PerCP-eFluor 710	44.189	ThermoFisher	46-3369-42	250
PD-1	BV785	EH12.2H7	BioLegend	329930	250
TCR Valpha7.2	BUV563	OF-5A12	BD Biosciences	749484	500
TCR Vd2	BUV615	B6	BD Biosciences	751368	15
TCRgd	BUV805	11F2	BD Biosciences	748532	500
TIGIT	BV480	Clone 741182	BD Biosciences	747843	250
TIM3	PE-CF594	7D3	BD Biosciences	565560	250
Viability	Live/Dead blue	–	Invitrogen	L23105	30 µl of 1:2000 diluted stock
B					
**Specificity**	**Fluorchrome**	**Clone**	**Vendor**	**Catalog number**	**Titer (ng/test)**
CD10	APC	HI10a	BioLegend	312210	1000
CD11b	PerCp-Cy5.5	ICRF44	BioLegend	301328	500
CD11c	eFluor450	Clone 3.9	ThermoFisher	48-0116-42	1000
CD123	BUV737	6H6	BD Biosciences	751839	70
CD14	Spark Blue 550	63D3	Biolegend	367148	100
CD141	BB515	1A4	BD Biosciences	565084	250
CD16	BUV496	3G8	BD Biosciences	612944	250
CD163	BUV661	GHI/61	BD Biosciences	741645	500
CD169 (Siglec-1)	BV421	7-239	Biolegend	346018	500
CD19	cFluor BYG710	HIB19	Cytek	SKU R7-20009	500
CD1c	Alexa Fluor 647	L161	BioLegend	331510	250
CD20	PE-eFluor610	2H7	ThermoFisher	61-0209-42	15
CD24	BV750	ML5	BD Biosciences	746890	500
CD27	APC-H7	M-T271	BD Biosciences	560222	250
CD3	Superbright 436	SK7	ThermoFisher	62-0036-42	250
CD303	PE-Cy7	201A	BioLegend	354214	125
CD33	BUV805	WM-53	BD Biosciences	742015	32
CD38	APC-Fire810	HIT2	BioLegend	303550	500
CD40	BUV395	5C3	BD Biosciences	565202	125
CD45	PerCP	2D1	BioLegend	368506	200
CD56	Superbright 436	TULY56	ThermoFisher	62-0566-42	250
CD73	BV650	AD2	BD Biosciences	742633	250
CD80	BV785	2D10	BioLegend	305238	500
CD86	PE-Fire810	IT2.2	Biolegend	305455	1000
CD95	PE-Cy5	DX2	BioLegend	305610	125
FcRL4	BV711	A1	BD Biosciences	747556	1000
FcRL5	PE	509f6	BioLegend	340304	500
HLA-DR	APC-R700	G46-6	BD Biosciences	565127	8
IgD	BV480	IA6-2	BD Biosciences	566138	125
IgG	BV605	G18-145	BD Biosciences	563246	125
IgM	BV570	MHM-88	BioLegend	314517	312.5
Ki67	BV510	B56	BD Biosciences	563462	750
PD-L1	PE-Dazzle594	29E.2A3	BioLegend	329732	1000
Viability	Live/Dead blue	–	Invitrogen	L23105	30 µl of 1:2000 diluted stock

*Custom conjugation available via Cytek.

Antibody conjugations used for panel 1 (A) and panel 2 (B), including clone number, vendor, catalog number and concentration in ng/test used in for the staining protocol described in section 5.

### 2.3 Equipment

The current method was developed for a Cytek^®^ Aurora (Cytek Biosciences Inc., Fremont, California) equipped with 5 lasers (355, 405, 488, 561, 640 nm) and 64 detectors. Details on the cytometer configuration, including the number of detectors per laser line and the bandwidth of the corresponding filters are presented in **Supplementary Table 1**.

## 3 Methods

### 3.1 Marker selection

For both panels, markers presented in a previously published method (5) were used as starting point, with selection of additional markers unique to the panels described here, based on the literature (8–18).

For both panels, CD45 was selected as a pan-leukocyte marker. In panel 1, the lineage markers CD19, CD123, CD14, and CD1c were included for exclusion of (lineage gate) of B cells, monocytes, and dendritic cells, which were investigated in panel 2. CD3 was selected as pan-T cell marker. TCRγδ and TCRVδ2 were included to identify γδ1/3 T cells (CD3^+^TCRγδ^+^TCRVδ2^-^) and γδ2 T cells (CD3^+^TCRγδ^+^TCRVδ2^+^) (5, 6, 19, 20). TCRVα24J18, TCRVα7.2, and CD161 were used for characterizing invariant natural killer T (iNKT) cells (CD3^+^TCRVα7.2^-^TCRVα24J18^+^) (6) and the following mucosal-associated invariant T (MAIT) cell subsets: MAIT cells (TCRVα7.2^+^CD8^+^CD161^+^) and CD8^-^ MAIT cells (TCRVα7.2^+^CD8^-^CD161^+^), in addition to TCRVα7.2^+^CD8^+^CD161^-^ cells, and TCRVα7.2^+^CD8^-^CD161^-^ cells (21–24). CD56 was added for gating on the natural killer T (NKT)-like cells within the CD3^+^non-MAIT/γδ/iNKT (CD3^+^iNKT-TCRVα7.2^-^TCRγδ^-^TCRVδ2^-^) cell compartment (CD3^+^CD56^+^) (25, 26). CD4, CD8, and CD27, CD45RA, CD28, CD95, and CCR7 were included in panel 1 to identify cytotoxic (CD3^+^CD8^+^) and helper (CD3^+^CD4^+^) T cells as well as their Naïve (CD45RA^+^CCR7^+^CD95^-^), central memory (TCM) (CD45RA^-^CCR7^+^CD28^+^CD27^+^), terminal effector memory re-expressing CD45RA (TEMRA) (CCR7^-^CD45RA^+^), and effector memory (EM) (CD45RA^-^CCR7^-^) subsets (5, 27–35). With the addition of CD27, CD28, and CD95, T cell subsets can be further divided into memory stem cell (TSCM) (CD45RA^+^CCR7^+^CD27^+^CD95^+^), early-like effector (CD45RA^-^CCR7^-^CD28^+^CD27^-^), early effector (CD45RA^-^CCR7^-^CD28^+^CD27^+^), intermediate effector (CD45RA^-^CCR7^-^CD28^-^CD27^+^), and terminal effector (CD45RA^-^CCR7^-^CD28^-^CD27^-^) (27, 29, 30, 33–35). Within CD4^+^ T cells, CXCR5, CD25, and FoxP3 were used to resolve regulatory T cells (Treg) (CD3^+^CD4^+^CXCR5^-^FoxP3^+^CD25^+^) (36) and memory T follicular helper cells (TFH) (CD3^+^CD4^+^CD45RA^-^CXCR5^+^) (37, 38). In the CD8^+^ T cell compartment, CXCR5 was used to identify CXRC5^+^ CD8 T cells (CD8^+^CXCR5^+^) (39, 40).

For the CD3^-^ population, CD56 and CD16 were included for the identification of the three classical subsets of peripheral NK cells: early NK cells (CD56^high^CD16^-^), mature NK cells (CD56^+^CD16^+^), terminal NK cells (CD56^-^CD16^+^) (41). CD127 was used to identify ILCs within the CD16^-^CD56^-^ cell population. CRTH2 and cKIT were added for subdivision of ILCs into ILC1 (CD16^-^CD127^+^CRTH2^-^cKIT^-^), ILC2 (CD16^-^CD127^+^CRTH2^+^cKIT^-^) and ILC3 (CD16^-^CD127^+^CRTH2^-^cKIT^+^) (42, 43).

In addition to the lineage markers described above, we included in panel 1 functional markers to assess activation, proliferation, and exhaustion of the cell types described so far. CD69 was included to assess activation of T cells and NK cells (44–46), CD39 was included as marker for activation and effector function of regulatory T cells (45–47), the immune checkpoint markers TIGIT, PD-1, CTLA-4, and TIM-3 were included to evaluate T cell exhaustion (48), LAG3 was used for evaluation of T cell exhaustion and effector function of NK cells (49, 50), and Ki67 was added as marker for proliferation potential. CD57 was included as a maturation marker for NK cells, to identify terminally differentiated NK cells with high cytotoxic and diminished replicative potential (51). Furthermore, NKG2A and NKG2C were included. The inhibitory receptor NKG2A is expressed on mature NK cells with high cytotoxic potential. Mature NK cells can differentiate into adaptive NK cells, capable of self-renewal. This differentiation is characterized by reduced expression of NKG2A and increased expression of NKG2C (52–54). NKp44 was included to asses activation of NK cells, ILCs and NKT-like cells (54, 55).

In panel 2, the lineage markers CD3 and CD56 were used to exclude T cells and NK cells (lineage negative gate), which are evaluated in panel 1. CD19 and CD20 were included to gate on B cells (CD20^+^CD19^+^), further separated into B cell subsets by the markers CD10, CD24, IgM, CD27, IgD, IgG, CD38 and PD-L1 to identify in the IgD^+^ compartment transitional B cells (IgD^+^CD10^+^CD24^high^) (10), marginal zone B cells (CD27^+^IgD^+^IgM^+^), naïve B cells (IgD^+^CD27^-^CD24^+^CD38^-^) (5, 25, 56) and IgD^+^CD24loCD38^^+^/^-^^ B cells, also described as regulatory B cells (B¬reg) (57, 58), which are thought to regulate TFH activity, specifically PD-L1^high^Bregs (IgD^+^CD24loCD38^+^/^-^PD-L1^high^) (59). From the IgD^-^ population, plasmablasts were identified as CD19^+^IgD^-^CD20^lo^CD38^high^ (60), memory B cells as CD19^+^CD20^+^IgD^-^CD27^+^, and a CD27^-^CD24^-^ population indicated as such (61, 62). With IgM and IgG, the following B cell populations were resolved: IgM^+^ B cells (IgM^+^IgG^-^), IgG^+^ B cells (IgM^-^IgG^+^) and IgM^-^IgG^-^ B cells (IgM^-^IgG^-^) (10), further subdivided into memory (CD24^+^) (63) and activated (CD38^+^) (64, 65) subsets.

From the lineage^-^CD19^-^ population, CD11c^-^ cells were identified using CD11c, and HLA-DR and CD123 were added to resolve basophils from the CD11c^-^ subset (HLA-DR^-^CD123^+^) (66). From the remaining lineage^-^CD19^-^ cells, myeloid cells were identified based on SSC and CD11c. Then, with use of CD1c, CD141 and CD303 the following dendritic cell (DC) populations were identified: conventional DC (cDC)2 (CD11c^+^CD141^+^), cDC1 (CD11c^-^/^+^CD141^+^) and plasmacytoid DC (pDC) (CD11c^-^CD303^+^) (9, 67, 68). From the remaining myeloid cells, we identified classical monocytes (CD14^+^CD16^-^), intermediate monocytes (CD14^+^CD16int), non-classical monocytes (CD16^+^CD14^+^) (5, 25). From the CD16^-^CD14^-^, early myeloid-derived suppressor cell (eMDSC) are identified as lineage^-^CD19^-^CD14^-^HLA-DR^-^CD11b^+^CD33^+^ (69).

Aside from the lineage markers described here, several functional markers were included in panel 2 for evaluation of activation and proliferation of the cell types considered in panel 2. CD40, CD24, CD73, FcRL4 and FcRL5 were used to assess B cell activation and differentiation (70–73). For macrophage activation CD80, CD86, CD169 and CD163 were included (74–78).

The marker combinations used for resolving the target populations and assessing their functional status as described above are summarized in **Table 1**.

### 3.2 Fluorochrome assignment

First, we applied the basic principles for panel design best practices as described elsewhere (79), by classifying markers as primary, secondary, and tertiary based on expression levels as depicted in **Supplementary Table 2**. Secondly, we used the Cytek Fluorochrome Guide for the 5 Laser Aurora for fluorochrome brightness classification. The classification was used as a guide to pair markers with low antigen density with brighter fluorochromes and markers with high antigen density with dimmer fluorochromes as much as possible. Assessment of expression levels and co-expression on different leucocyte subsets was based upon the literature. Thirdly, we used Cytek Full Spectrum viewer to identify fluorochromes with unique spectra to accommodate all the markers in each panel thereby minimizing fluorochrome pairs with high Similarity Indices (5). Normalized spectra of fluorochromes used for the current method are shown in **Supplementary Figure 1**. The compatibility of the selected fluorochromes was evaluated for both panels by calculating the Similarity Index Matrix in the SpectroFlo software and is displayed by a heatmap (**Supplementary Table 3**). The Similarity Index represents the similarity between two spectra. A value of ‘1’ indicates that two spectra are identical, a value of ‘0’ means that two spectra are unique. Fluorochromes with a Similarity Index of < 0.99 can be properly unmixed, so only combinations with a Similarity Index below 0.99 were included in the panels. Furthermore, the Complexity Index, a measure of the complexity of the total number of fluorochromes in the panel, was kept as low as possible. None of the fluorochrome combinations in either of the panels reached a Similarity Index of 0.99 or higher. Fluorochrome pairs with the Similarity Indices > 0.90 were SuperBright 436 and BV421 (0.97, both panels), PE-eFluor 610 and PE-Dazzle594 (0.96, panel 2), SuperBright 436 and eFluor 450 (0.94, both panels) and PE and cFluor YG584 (0.92, panel 1). The Complexity Index for panel 1 was 32.59 and 25.94 for panel 2. Next, we made an effort to avoid placing highly expressed antigens in close proximity to co-expressed antigens with low expression, both within columns (indicating primary excitation laser) and within rows (indicating similar emission wavelength), limiting both these sources of spread (to minimize cross laser excitation where both fluorochromes are excited by different lasers but emit in similar spectral regions). Subsequently, we generated the Spillover Spread Matrix (SSM) using PBMCs stained with CD4-conjugated fluorochromes selected as described above, to predict and minimize areas of spread and to guide further marker-fluorochrome assignments considering co-expression and expression levels. SSM values > 10 were for PerCP-eFluor710 into cFluor BYG710 (11.18), PE-Cy5 into cFluor BYG710 (12.77) and into PE-Alexa Fluor 700 (12.09) and APC-R700 into cFluor BYG710 (12.87) in panel 1 (**Supplementary Table 4A**). In panel 2 none of the SSM values were > 10, the highest spread coming from APC-R700 into cFluor BYG710 (8.34) (**Supplementary Table 4B**). Next, we evaluated the staining pattern for each of the selected fluorochromes for unmixing with only the respective Reference Controls (RCs) and autofluorescence (AF) versus unmixing with the total of 36 or 33 RCs included in the panel. Final marker-fluorochromes assignment was done considering all criteria described above.

### 3.3 Titrations

Some of the antibodies used for the panels presented here, have been titrated previously (5, 9, 80, 81). When available, and since Cytek Aurora’s performance is matched via Cytek Assay Settings (CAS), titers were selected from previously published work for all antibody-fluorochrome combinations that are compatible with the eBioscience Foxp3/Transcription Factor Staining Buffer Set, with the consideration that antibody lot variations would be minor and could be adjusted once the panel is completed. An overview of titers selected from previously published work is presented in **Supplementary Table 5**. All other antibodies were titrated on cryopreserved PBMCs from healthy donors, or cryopreserved tonsil cells using 1*10^6 cells/test. All samples were stained in a total volume of 150 µl, consisting of 5 µl True-Stain Monocyte Blocker, 5 µl BD Pharmingen Human Fc Block and 10 µl BD Horizon Brilliant Stain buffer to match the multicolor (MC) staining procedure, supplemented with wash/stain buffer to reach 150 µl, as was done for the MC samples. All samples were fixed and permeabilized using the eBioscience Foxp3/Transcription Factor Staining Buffer Set, matching the procedure for the MC sample, as described in the staining protocol in section 3.6. Antibodies were tested in 2-fold dilutions ranging from 15 ng/test to 1000 ng/test or in some cases from 8 ng/test to 500 ng/test. Concatenated files were generated for titration evaluation. The optimal titers were selected based on stain index calculated from the Mean Fluorescence Intensity (MFI), and considering non-specific binding to monocytes, spread and brightness of the negative population, and percent of positive cells (82). Staining pattern and non-specific binding were evaluated by plotting each marker vs SCC. The results of the titration experiments are presented in **Supplementary Figure 2**. Results for all markers titrated for the current method are presented. An overview of all reagents, selected titers and vendor information for the current method is presented **Table 2**.

### 3.4 Panel optimization

The full panel was tested and resolution of each of the markers was evaluated by comparing the RC to the corresponding marker in the MC sample (**Supplementary Figure 3**). For markers with high SSM values, the impact of the spread was assessed by visual evaluation of those combinations (**Supplementary Figure 4**), and did not indicate negative impact on the performance of the panels. Adjustments were made to fluorochrome selection and assignment, titers, and to the staining protocol over several iterations of panel testing until resolution of all included markers was sufficient. After the first full test staining of panel 1, a loss of resolution in the MC sample was observed for negative selection on the lineage markers (SuperBright 436), CD39 BUV661 and LAG3 BV711. The initially selected titers for these markers were increased by 2-fold in the next panel iteration, solving this problem as shown in **Supplementary Figure 5A**. The initial selection of fluorochromes allowed for the addition of two more fluorochromes without increasing the Complexity Index and the spread unacceptably. Therefore, after the second full test staining, TCR Vα24-Jα18 and NKp44 were added on cFluor BYG710 and on PerCP-eFluor710 respectively. Fluorochrome selection for these two antibodies was done as described in the section on fluorochrome assignment and evaluation of the marker resolution was done as described above. After the second panel iteration, a loss of resolution was observed in the MC sample for TIGIT, FoxP3, CD56, CD27 and CD127 which was solved by increasing the titers by two-fold as shown in **Supplementary Figure 5A**. Furthermore, a loss of resolution of CD161 was observed in γδ T cells and NK cells. Sequential 10 minute incubation with the TCR antibodies (TCRγδ, TCRVα7.2, TCR Vα24-Jα18), CCR7 and CXCR5 in 3 separate steps before addition of the remaining antibodies, sufficiently improved the CD161 resolution, as is shown on γδ T cells in **Supplementary Figure 5B**. After the third and final panel iteration the titers of TCRγδ BUV805, TCRVα7.2 BUV536, and NKG2A APC were increased by 2-fold to improve the resolution. An overview of differences in resolution of the markers mentioned above before and after changes described for these three panel iterations are shown in **Supplementary Figure 5A**. In panel 2, CD20 was moved from BUV395 to PE-eFluor610 as resolution of BUV395 for this marker was not acceptable. This also enabled the addition of CD40 conjugated to BUV395 to the panel. In the second panel iteration, CD19 Spark NIR was exchanged for CD19 cFluor BYG710 as the resolution of the initially selected Spark NIR was insufficient. Furthermore, titers of the following markers were increased by two-fold to solve a loss of resolution: CD11b, CD11c, CD45, and CD27 as shown in **Supplementary Figure 5C**. Lastly, we observed FcRL5 staining on monocytes. Addition of a 10 minute incubation step with only IgG prior to the staining with the rest of the antibodies solved the artifact possibly by inhibiting the binding of FcRL5 antibody to the IgG that is inherently bound to the monocytes (83), as shown in **Supplementary Figure 5D**. The final protocol established after panel design and evaluation of the marker resolution is presented below.

### 3.5 Thawing of PBMCs and tonsil samples

Timing: 1–4 hours, depending on the number of samples and the method for cell counting (manual or automated)

1. Prepare thawing medium: RPMI 1640 supplemented with 10% heat-inactivated FBS (Store at 4 °C for up to 2 months).
2. Warm thawing medium in water bath at 37 °C. If the volume of the medium is large, warm at least 30 minutes in advance.
3. Thaw cryovials in a water bath (37 °C) for 1–2 minutes until a small clump of ice is remaining.
4. Place the 1 mL cell suspension the cryovial into a 15 ml tube, labeled with sample identification. Aspirate 9 ml of warm medium, add 1 ml to the emptied cryovial to rinse the vial and add the remaining 8 ml to the 15 ml tube, dropwise, while gently swirling the tube. Add the 1 mL medium from the cryovial to the 15 ml tube.
5. Spin at 350 x *g* for 10 minutes at 4 °C.
6. Carefully decant the supernatant and gently resuspend the pellet in 10 ml warm thawing medium.
7. Take a 10 µL aliquot for cell counting, dilute 1x with Trypan blue and count live and dead cell using a hemocytometer or automated cell counter.
8. Repeat step 4 and 5 and leave samples on ice until staining.

Note: To avoid artifacts in the data introduced by low cell viability or large differences in cell recoveries, it is important to count cells and calculate the cell viability.

Critical: Once thawing of the samples is started, samples need to be processed as fast as possible to maintain cell viability.

### 3.6 Flow cytometry staining protocol

#### 3.6.1 Preparing viability dye, buffers, and antibody mixes

Timing: 2 hours

1. Thaw an aliquot of Live/Dead Blue staining.
2. Prepare the required amount of Live/Dead blue staining by diluting Live/Dead Blue fixable viability dye 2000x in PBS.
3. Prepare the wash/stain buffer by adding 2.5 g BSA to 500 ml PBS (0.5% BSA) and mixing until fully dissolved. Store at 4 °C until use for up to 1 month.
4. Prepare the antibody mixes fresh on the day of use, as follows according to **Table 3**:
  - Prepare the staining buffer: combine human Fc block, True-Stain Monocyte block, Brilliant Staining Buffer and wash/stain buffer for panel 1 and panel 2 separately in 15 ml tubes. Prepare enough reagent for all multicolor samples, unstained samples and Reference Controls (RCs).
  - Prepare the TCR antibody mix: combine all four TCR antibodies in a 0.5 ml Eppendorf tube, resuspend to combine.
  - Prepare panel 1 surface staining mix: combine all remaining surface staining antibodies of panel 1 except for the Live/Dead Blue fixable viability stain, anti-CCR7 and anti-CXCR5 in Brilliant Staining buffer in a 15 ml tube, resuspend to combine. Alternatively, this step can be done or finalized during the incubations steps for the viability staining (section 3.6.3) and surface staining with TCR and cytokine antibodies (3.6.4).
  - Prepare panel 2 surface staining mix: combine all surface staining antibodies of panel 2 except for the Live/Dead Blue fixable viability stain, and anti-IgG in Brilliant Staining buffer in a 15 ml tube, resuspend to combine. Alternatively, this step can be done or finalized during the incubation steps for the viability staining (section 3.6.3) and surface staining with IgG (section 3.6.5).
  - Prepare FoxP3 Fixation/Permeabilization working solution by adding 1 part Fixation/Permeabilization concentrate to 3 parts Fixation/Permeabilization diluent according to the manufacturer’s instructions. Prepare enough reagent for all multicolor samples, unstained samples and RCs for both panels. Alternatively, this reagent can be prepared during step 21–23 below.
  - Prepare FoxP3 Permeabilization buffer by diluting 10X permeabilization buffer 1:10 in Ultrapure water according to the manufacturer’s instructions. Prepare enough reagent, at least 750 µL for each multicolor sample, unstained sample and RC for both panels. Alternatively, this reagent can be prepared during the fixation/permeabilization of the samples (section 3.6.6.).
  - Prepare panel 1 intracellular staining mix: combine FoxP3 Permeabilization buffer with all three intracellular staining antibodies.
  - Prepare panel 2 intracellular staining mix: combine FoxP3 Permeabilization buffer with anti-KI-67. Alternatively, intracellular staining mixed can be prepared during the fixation/permeabilization of the samples (section 3.6.6.).
  - Keep all mixes stored at 4 °C and protected from light until use.
5. Prepare plate layouts for all plates.

**Table 3:** Overview of constituents of reagent mixes for panel 1 and 2.

A.				
Panel	Antigen	Fluorochrome	Staining	µl/test (= 150 µl)
Both	Viability	Live/Dead blue	1. Viability	0.5
	PBS			1000
	BD Pharmingen™ Human Fc Block™		2. Staining buffer	5
	True-Stain Monocyte Blocker™		2. Staining buffer	5
	BD Horizon™ Brilliant Stain buffer		2. Staining buffer	10
	FACS Buffer		2. Staining buffer	33.48
1	TCR Vd2	BUV615	3. TCR antibody mix	0.06
1	iNKT (TCR Vα24-Jα18)	cFluor BYG710	3. TCR antibody mix	0.71
1	TCR Valpha7.2	BUV563	3. TCR antibody mix	1.88
1	TCRgd	BUV805	3. TCR antibody mix	1.88
1	CCR7	BV421	4. Chemokine receptors	3.00
1	CXCR5	BV750	4. Chemokine receptors	0.38
	Brilliant Staining buffer		5. Surface staining mix	10.00
1	CD45RA	BUV395	5. Surface staining mix	0.19
1	CD4	cFluor YG584	5. Surface staining mix	0.38
1	CD16	BUV496	5. Surface staining mix	0.47
Both	CD95	PE-Cy5	5. Surface staining mix	0.47
1	TIM3	PE-CF594	5. Surface staining mix	0.63
1	CD25	PE-AlexaFluor700	5. Surface staining mix	0.75
1	CD57	Fitc	5. Surface staining mix	0.94
1	TIGIT	BV480	5. Surface staining mix	0.94
1	CD127	APC-R700	5. Surface staining mix	1.50
1	CD28	BV650	5. Surface staining mix	1.50
1	CD3	Spark Blue 550	5. Surface staining mix	1.50
Both	CD45	PerCP	5. Surface staining mix	1.50
1	NKG2c (CD159c)	PE	5. Surface staining mix	1.50
1	CD14	Superbright 436	5. Surface staining mix	1.88
1	CD39	BUV661	5. Surface staining mix	1.88
1	PD-1	BV785	5. Surface staining mix	1.88
1	CD69	BUV737	5. Surface staining mix	3.00
1	cKIT	BV605	5. Surface staining mix	3.00
1	CRTH2	PE-Cy7	5. Surface staining mix	3.00
1	NK2GA (CD159a)	APC	5. Surface staining mix	3.00
Both	CD27	APC-H7	5. Surface staining mix	3.75
1	CD123	Superbright 436	5. Surface staining mix	3.75
1	CD56	Spark 685 NIR	5. Surface staining mix	3.75
1	CD8	APC-Fire810	5. Surface staining mix	3.75
1	NKp44 (CD336)	PerCP-eFluor 710	5. Surface staining mix	3.75
1	CD161	eF450	5. Surface staining mix	7.50
1	CD19	Superbright 436	5. Surface staining mix	7.50
1	CD1c	Superbright 436	5. Surface staining mix	7.50
1	Lag3	BV711	5. Surface staining mix	7.50
1	CTLA-4	Alexa Fluor 647	6. Intracellular stainig mix	0.94
1	Foxp3	BB700	6. Intracellular stainig mix	6.00
Both	KI67	BV510	6. Intracellular stainig mix	1.88
	1x Fixation/Permeabilization buffer			141.19
B.				
Panel	Antigen	Fluorochrome	Staining	µl/test (= 150 µl)
Both	Viability	Live/Dead blue	1. Viability	0.5
	PBS			1000
	BD Pharmingen™ Human Fc Block™		2. Staining buffer	5
	True-Stain Monocyte Blocker™		2. Staining buffer	5
	BD Horizon™ Brilliant Stain buffer		2. Staining buffer	10
	FACS Buffer		2. Staining buffer	42.45
2	IgG	BV605	3. IgG	1.88
	Brilliant Staining buffer		4. Surface staining mix	3.75
2	HLA-DR	APC-R700	4. Surface staining mix	0.06
2	CD33	BUV805	4. Surface staining mix	0.12
2	CD14	Spark Blue 550	4. Surface staining mix	0.19
2	CD20	PE-eFluor610	4. Surface staining mix	0.23
2	CD123	BUV737	4. Surface staining mix	0.26
Both	CD16	BUV496	4. Surface staining mix	0.47
2	CD303	PE-Cy7	4. Surface staining mix	0.47
Both	CD95	PE-Cy5	4. Surface staining mix	0.47
2	IgD	BV480	4. Surface staining mix	0.47
2	CD19	cFluor BYG710	4. Surface staining mix	0.63
2	CD141	BB515	4. Surface staining mix	0.94
2	CD73	BV650	4. Surface staining mix	0.94
2	CD11b	PerCp-Cy5.5	4. Surface staining mix	1.88
2	CD163	BUV661	4. Surface staining mix	1.88
2	CD24	BV750	4. Surface staining mix	1.88
2	CD3	Superbright436	4. Surface staining mix	1.88
2	CD40	BUV395	4. Surface staining mix	1.88
2	IgM	BV570	4. Surface staining mix	2.34
2	CD38	APC-Fire810	4. Surface staining mix	2.48
2	PD-L1	PE-Dazzle594	4. Surface staining mix	3.75
2	CD169 (Siglec-1)	BV421	4. Surface staining mix	3.75
2	CD1c	Alexa Fluor 647	4. Surface staining mix	3.75
Both	CD27	APC-H7	4. Surface staining mix	3.75
Both	CD45	PerCP	4. Surface staining mix	3.75
2	CD56	Superbright436	4. Surface staining mix	3.75
2	CD80	BV785	4. Surface staining mix	3.75
2	CD86	PE-Fire810	4. Surface staining mix	3.75
2	FcRL4	BV711	4. Surface staining mix	3.75
2	CD10	APC	4. Surface staining mix	7.50
2	CD11c	eFluor450	4. Surface staining mix	7.50
2	FcRL5	PE	4. Surface staining mix	7.50
Both	KI67	BV510	5. Intracellular stainig mix	1.88
	1x Fixation/Permeabilization buffer			148.13

All reagents used for the staining protocol described in section 5 are listed here. Reagent volumes required for staining of one sample are presented in µl.

#### 3.6.2 Preparing samples for staining

Timing: 1 hour, depending on the number of samples

1. Spin samples at 350g for 10 minutes. This centrifugation step and all following centrifugations are done at RT.
2. Carefully decant the supernatant and dilute the pellet to 10*10^6 cells/ml by gently resuspending in wash/stain buffer.
3. Place 1*10^6 cells/sample (100 µL) in plate 1 for panel 1 and in plate 2 for panel 2, also include unstained samples on a third plate. Plate samples for RCs on separate plates, one for each panel. For some RCs beads can be used. For both panels, RCs on cells and beads have been tested and evaluated, the results can be found in **Supplementary Table 6**. Please note that UltraComp eBeads should not be treated with Brilliant Stain buffer. It is recommended to leave one row empty between each row of cells or beads for RCs to avoid contamination. For these panels, experiments were optimized in plates, alternatively 5 ml round bottom tubes can be used. In that case, tubes should be labelled adequately and the washing volume should be increased.
4. Spin plate at 450 x *g* for 5 minutes.
5. Aspirate supernatant, add 150 µL PBS to wash the samples.
6. Spin plate at 450 x *g* for 5 minutes. Aspirate supernatant.

Note: Autofluorescence extraction is essential for proper unmixing of spectral flow cytometry data. This requires the acquisition of unstained samples. Therefore, unstained samples have to be included, preferably one unstained sample for each donor. If this is not possible, an unstained sample should be included for each cell type or sample type where the autofluorescence is different from your controls, especially if there are multiple autofluorescence spectra within one sample type. The unstained controls can then be used to extract the autofluorescence spectra and improve the unmixing accuracy (84).

Note: For functional markers or markers with low expression levels (e.g., Ki67, FcRL4, FoxP3), it may be necessary to include stimulated cells or patient samples as RC, to ensure sufficient levels of markers expression and to avoid unmixing errors introduced when the fluorescence in the MC sample is brighter than in the RC. An unstained control of the selected RC sample should also be included to correct for potential differences in autofluorescence. In the current study, tonsil samples were used as RCs for some of the markers as shown in **Supplementary Table 6**.

Note: To reduce the number of samples per experiment, RC samples can be stained and acquired on a separate day prior to the staining and acquisition of the actual samples. RC samples can be re-used for following experiments (85), as long as the cytometer does not require major maintenance between acquisition of RCs and MC samples, and lot numbers of the antibodies are the same for RCs and MC samples.

#### 3.6.3 Viability dye staining

Timing: 30 minutes

7. Add 30 µL of 2000x diluted Live/Dead Blue staining to each MC sample of both panels and the corresponding RC and 30 µL PBS to all unstained samples and remaining RCs. Resuspend pellet carefully and incubate for 20 minutes at RT in the dark.
8. After 20 min incubation, add 120 µL wash/stain buffer to all MC samples, unstained samples and RCs, and spin plate at 450 x *g* for 5 minutes. Aspirate supernatant.
9. Add 150 µL wash/stain buffer to all MC samples, unstained samples and RCs of panel 2. Store plates at 4 °C, protected from light, and continue with panel 1.

Note: To ensure sufficient marker expression it might be necessary to treat the RC for the viability dye before staining. To do so, split the RC sample in half, place one half of the sample in a 65 °C water bath for 5 minutes to induce apoptosis. Cool the heat shocked fraction shortly on ice and mix with the untreated fraction for a RC with approximately 50% death cells.

#### 3.6.4 Surface staining panel 1

Timing: 2–3 hours depending on the number of samples

10. Add 50.11 µL of the staining buffer to all MC samples, unstained samples and RCs, but not to the RCs on UltraComp eBeads, these should not be treated with brilliant stain buffer. Add 50.11 µL wash/stain buffer to those RCs instead.
11. Add 4.51 µL of the TCR mix to all MC samples, 4.51 µL wash/stain buffer to unstained samples and all SSC, except for the SSC of the TCR antibodies, which should all receive the volume of the respective antibody as shown in **Table 3**. Incubate for 10 min at RT in the dark.
12. Add the anti-CCR7 to all MC samples and the corresponding RC. Resuspend carefully and incubate for 10 min at RT in the dark.
13. In the meantime, start with staining of the RCs for the surface staining of panel 1 according to prepared plate layout and the antibody volumes presented in **Table 3**.
14. Add the anti-CXCR5 to all MC samples and the corresponding RC. Resuspend carefully and incubate for 10 min at RT in the dark.
15. Add 88.63 µL of panel 1 surface staining mix to all MC samples, an equal volume of wash/stain buffer to unstained samples and RCs. Resuspend carefully and incubate for 30 min at RT in the dark.
16. Finish RCs of panel 1 and incubate for 30 min at RT in the dark.
17. In the meantime, continue with surface staining of panel 2 (section 3.6.5, step 26).

#### 3.6.5 Surface staining panel 2

Timing: 2–3 hours depending on the number of samples

18. Spin plates at 450 x *g* for 5 minutes.
19. Add 62.45 µL staining buffer to all MC samples, unstained samples and RCs, but not to the RCs on UltraComp eBeads, these should not be treated with brilliant stain buffer. Add 50.11 µL FACS buffer to those RCs instead.
20. Add the anti-IgG for all MC samples and the corresponding SSC. Resuspend carefully and incubate for 10 min at RT in the dark.
21. In the meantime, start with staining of the RCs for the surface staining of panel 2 according to prepared plate layout and the antibody volumes presented in **Table 3**.
22. Add 85.68 µL of panel 2 surface staining mix to all MC samples, an equal volume of wash/stain buffer to unstained samples and RCs. Resuspend carefully and incubate for 30 min at RT in the dark.
23. Finish SSC of panel 2 and incubate for 30 min at RT in the dark.

#### 3.6.6 Fixation/permeabilization

24. After 30 minutes of incubation with surface staining (sections 3.6.4. and 3.6.5.), spin plates at 450 x *g* for 5 minutes.
25. Aspirate supernatant, wash with 150 µL wash/stain buffer and spin plates at 450 x *g* for 5 minutes.
26. Repeat step 19.
27. Add 150 µL of FoxP3 Fixation/Permeabilization working solution to all MC samples, unstained samples and RCs. Carefully and thoroughly resuspend the pellet, avoiding production of bubbles, and incubate for 30 min at RT in the dark.
28. Spin plates at 800 x *g* for 5 minutes. It is essential to increase relative centrifugal force to 800 x *g* to ensure adequate precipitation of fixated/permeabilizated cells which are lighter than live cells.
29. Aspirate supernatant, wash with 150 µL FoxP3 Permeabilization buffer and spin plates at 800 x *g* for 5 minutes.
30. Repeat step 29.
31. Pause here if needed: aspirate supernatant, resuspend all samples in 150 µL FoxP3 Permeabilization buffer and store at 4 °C for up to 24 hours before continuing with intracellular staining (section 3.6.7.). Spin plates at 800 x *g* for 5 minutes prior to step 32.

#### 3.6.7 Intracellular staining

Timing: 1–2 hours depending on the number of samples

32. Aspirate supernatant and add 150 µL of panel 1 intracellular staining mix to all MC samples of panel 1, 150 µL of panel 2 intracellular staining mix to all MC samples of panel 2, 150 µL FoxP3 Permeabilization buffer 1to all unstained samples and RCs and the corresponding antibodies to the RCs. Carefully resuspend and incubate for 45 min at RT in the dark.
33. In the meantime, label 1 ml small volume sampling tubes for MC samples, unstained samples and RCs of both panels. Alternatively, samples can be kept in the plate for automated sampling at the cytometer.
34. Spin plates at 800 x *g* for 5 minutes.
35. Aspirate supernatant, wash with 150 µL FoxP3 Permeabilization buffer and spin plates at 800 x *g* for 5 minutes.
36. Repeat step 35.
37. Aspirate supernatant, resuspend samples in 75 µL wash/stain buffer, and transfer contents to small volume sampling tubes. Add an additional 75 µL wash/stain buffer to collect all remaining sample and add to corresponding small volume sampling tubes.
38. Store samples at 4 °C in the dark for up to 24 hours until acquisition.

Note: Indications for timing presented at each step of the methods is the total time needed for that section, including incubation steps. During incubation steps, reagent preparation, preparation of antibody mixes and RC samples can be done, reducing the duration of the protocol.

### 3.7 Instrument QC and sample acquisition

Daily instrument quality control (QC) was run on the prior to sample acquisition using SpectroFlo^®^ QC beads. Samples were measured with default Cytek Assay Settings (CAS) provided by the instrument manufacturer. Gain settings for forward scatter (FSC) and sideward scatter (SSC) and the threshold were adjusted to capture all the relevant events on scale and minimizing the collection of debris. The current method is optimized for the Cytek Aurora 5 laser spectral flow cytometer. If a different instrument is used, verify compatibility of all fluorochrome-antibody pairs, and optimize the panel for the instrument of choice.

### 3.8 Unmixing of the MC samples

The SpectroFlo software V3.2.1 was used to unmix the MC samples, using beads, PBMCs or tonsil samples as RC, as shown in **Supplementary Table 6**. Marker resolution in the full samples was evaluated as described by Ferrer-Font et al. (86). In short, debris, doublets, dead cells and events acquired during an unstable flow period were manually removed and data was plotted in N x N plots, showing every fluorochrome versus all other fluorochromes in the panels. Unmixing was evaluated by visual inspection of the N x N plots and spillover correction was applied if needed.

Note: Spillover correction is generally acceptable up to 3%. If more than 3% correction is required, return to unmixing and identify the source of the issue. Check whether the reference control was performed on the correct sample type (beads vs. PBMCs vs. tonsil), verify lot number matching for tandem dyes, and assess whether autofluorescence is contributing to unmixing errors.

### 3.9 Data preprocessing and batch normalization

Unmixed data were transferred to the OMIQ platform for data pre-processing and analysis. The data was first scaled to make sure the negative populations were distributed around 0 and positive populations were on scale for all relevant channels. Then, the automated quality control algorithm PeacoQC (87) was used to clean the data with the following default settings: all files, all features, no prefilter, Maximum allowed median absolute deviations 6, Isolation tree gain limit 0,55, minimum number of consecutive bins 5, Remove zeros before peak detection, remove events with marginal values, generate plots and time units per visualization bin 100. Removal of debris, doublets, dead cells and CD45- cells, was done by manual gating.

As the samples were acquired in multiple batches, each batch contained a PBMC sample from the same healthy donor, which was used as the reference sample for batch correction. Batch correction is done to adjust for changes in the data that are introduced by the treatment and/or acquisition of the samples rather than the biology itself. Batch correction was applied by CytoNorm (88), on clusters generated by FlowSOM (89) run on all Live, CD45+, lineage- single cells in all the samples, with all fluorescent features except Live/Dead Blue, Superbright463 (lineage- channel) and autofluorescence and the following settings for both panels: xdim 10, ydim 10, rlen (number of training iterations) 10, distance metric Euclidean, run elbow metaclustering and random seed 5544. CytoNorm was then applied on the FlowSOM metaclusters-elbow on the same samples and same fluorescence parameters as the FlowSOM, with following settings for both panels: number of quantities 101, Batch metadata field “Batch” and Controls metadata field “Reference”. From then on, only the patient and healthy donor samples were included for downstream data processing, the reference samples were left out.

### 3.10 Data analysis

A targeted manual gating strategy was applied on the normalized data for both panels, to verify the resolution of all target populations in the panels. To assess the robustness of our manual gating strategy, seven cytometry experts performed the gating strategy described in this paper on all healthy donor samples included in the study (n = 8). For each target population, the percentage of parent was exported and compared between observers. Percentage of parent was chosen over absolute cell counts to avoid the accumulation of variation across sequential gating steps in populations requiring extensive hierarchical gating. For each target population, the coefficient of variation (CV%) was calculated on the percentage of parent values across the seven observers. CV% was selected as the variation metric because, unlike absolute measures of dispersion (e.g., standard deviation), it expresses variability relative to the mean and is therefore not confounded by the absolute size of the target population.

Next we applied unsupervised analysis to use the full potential of the panels developed in the present study. To this end, the data were subsampled to equal numbers across all samples, adjusted to the lowest available number of cells (54019 or 94364 Live, CD45+, lineage- single cells per sample (panel 1 and panel 2)), and a dimension reduction algorithm (Uniform Manifold Approximation and Projection (UMAP) (90)) was applied to the data on all samples, and the lineage markers: CD45RA, CD16, TCRVα7.2, TCRVδ2, TCRγδ, CCR7, CD161, cKIT, CD28, CXCR5, FoxP3, CD4, CD95, CD25, TCR Vα24-Jα18, CRTH2, CD56, CD127, CD27, CD8, NKG2A and NKG2C (panel 1) and CD16, CD123, CD11c, IgD, IgM, IgG, CD141, CD14, CD20, CD19, CD10, CD1c, HLA-DR, CD27 and CD38 (panel 2). The following default settings suggested by OMIQ were applied: Neighbors 15, Minimum distance 0.4, Components 2, Metric “Euclidian”, Learning Rate 1, Epochs 200, Random seed 8277 (panel 1) or 2523 (panel 2), Embedding Initialization “spectral”. Phenograph was applied for unsupervised clustering of the data on the same samples and features applied in the UMAP and the following default settings suggested by OMIQ: Nearest Neighbors 20, Nearest Neighbors algorithm “Annoy”, distance metric “Euclidean”, run elbow metaclustering, Clustering method “Leiden”, Leiden Seed 9742 (panel1) and 147 (panel 2). Phenograph clusters were annotated using clustered heatmap analysis, ran using the following default settings suggested by OMIQ: Row distance Euclidean, Row linkage ward, using a heatmap projecting the normalized MFI’s of the lineage markers in each cluster. To further assess unsupervised analysis performance at the lineage level, additional dimensionality reduction was applied to subsets of the data corresponding to the T cell, unconventional T cell, and B cell lineages (panel 1 and panel 2, respectively), using optimized t-Distributed Stochastic Neighbor Embedding (opt-SNE) (91) with the following default settings suggested by OMIQ: Max iterations 1000, opt-SNE End 5000, Perplexity 30, Theta 0.5, Components 2, Random Seed 6389 (conventional T cells), 7571 (unconventional T cells), and 4776 (B cells), and Verbosity 25. The same markers were included as described for the generation of the UMAP, except for the B cells, for which only IgD, IgM, IgG, CD24, CD45, CD20, CD19, CD10, HLA-DR, CD27, and CD38 were used. The resulting opt-SNE projections were overlayed with the Phenograph cluster allocations and with the manual gating annotations of the corresponding lineage, allowing direct comparison between the unsupervised and manual gating approaches at the subset level.

## 4 Results

### 4.1 Data preprocessing

**Figure 1:**
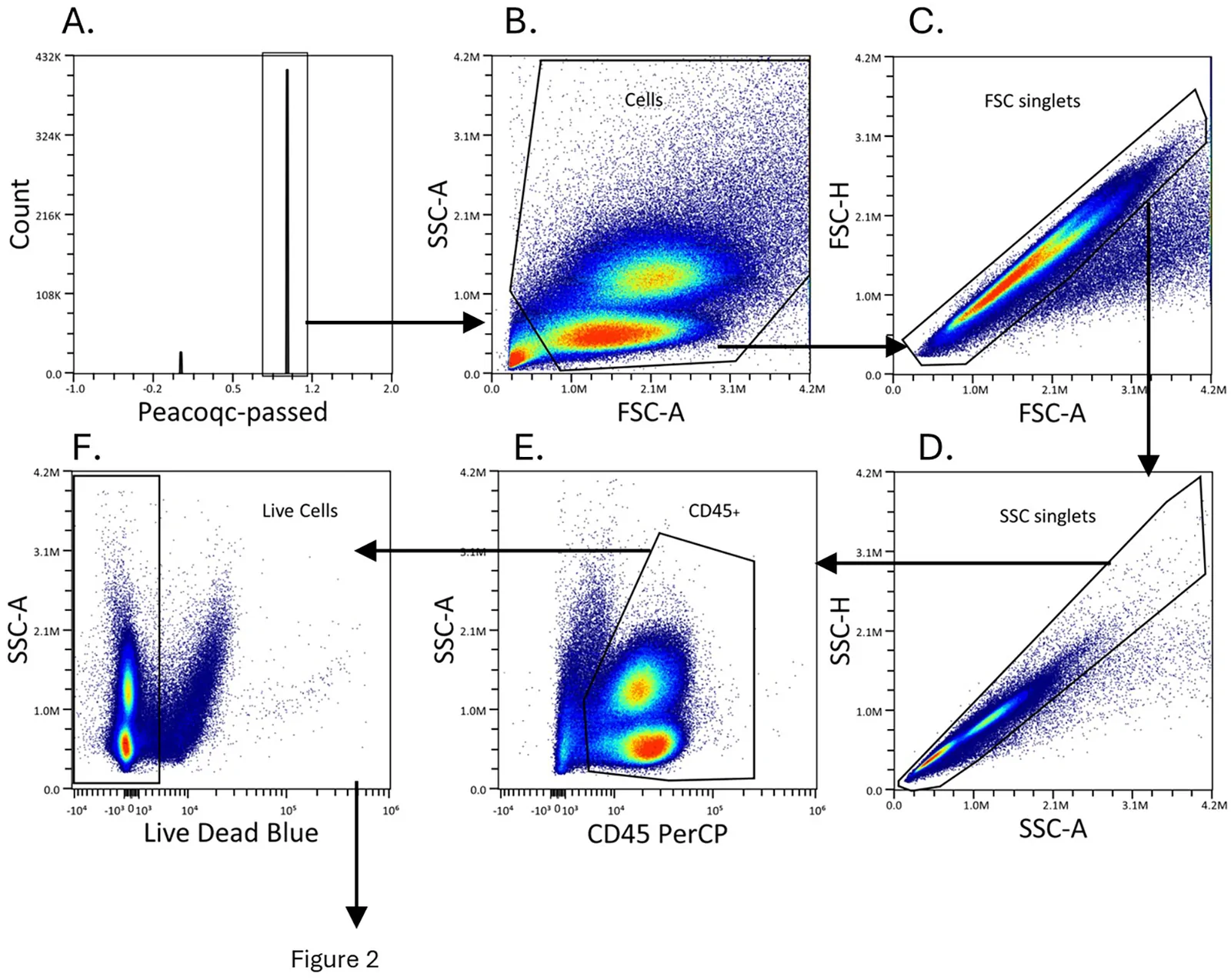
Manual cleaning of the data. Manual cleaning was done by gating on events that passed the automated quality control algorithm (PeacoQC) **(A)**, gating out debris **(B)**, selecting single cells on the Forward Scatter (FSC) **(C)** and the Sideward Scatter (SSC) **(D, E)** gating on the CD45^+^, and **(F)** viable cells. Data shown are of a representative sample of a healthy donor. All flow cytometry plots shown here and in following figures were generated In OMIQ (Dotmatics).

Data were automatically cleaned using the PeacoQC algorithm, followed by manually excluding debris, doublets, dead cells and CD45^-^ cells as shown in **Figure 1**.

### 4.2 Manual gating

A full manual gating strategy was then applied for both panels to verify the resolution of all target populations in the panels as follows.

**Figure 2:**
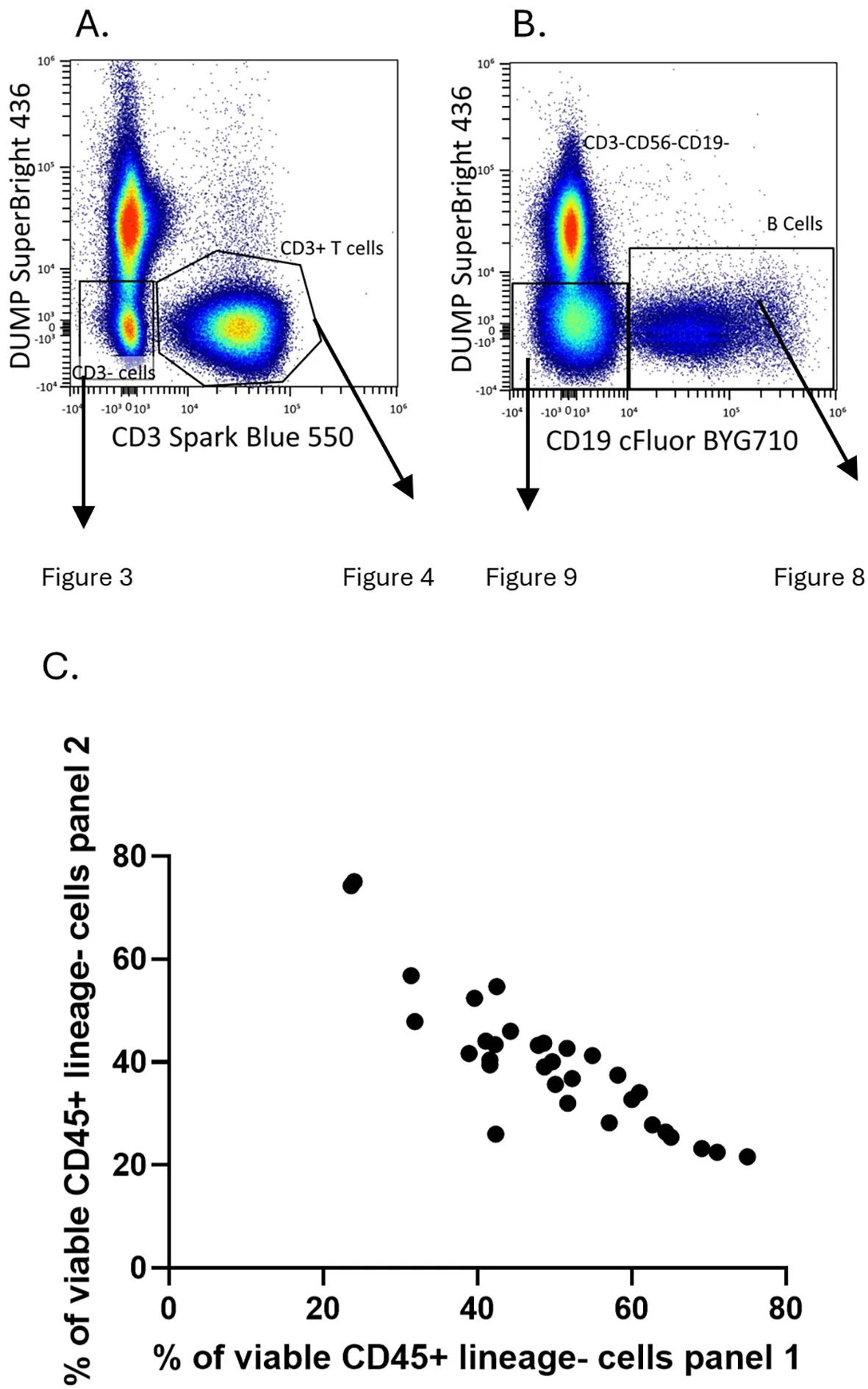
Selection of lineage^-^ events in panel 1 **(A)** and panel 2 **(B)** from viable, CD45^+^ single events that passed quality control (QC). **(C)** The correlation between the proportion of lineage negative cells in the same sample for both panels is presented as a % of viable, CD45+ single cells.

Lineage^-^ cells were selected for both of the panels, excluding B cells, monocytes and DCs (CD19^+^, CD14^+^, CD123 and/or CD1c^+^ events) from the data of panel 1 and excluding T cells and NK cells (CD3^+^ and/or CD56^+^ events) from the data of panel 2, as shown in **Figures 2A, B**. The correlation between the proportion of lineage negative cells in the same sample is presented in **Figure 2C**.

**Figure 3:**
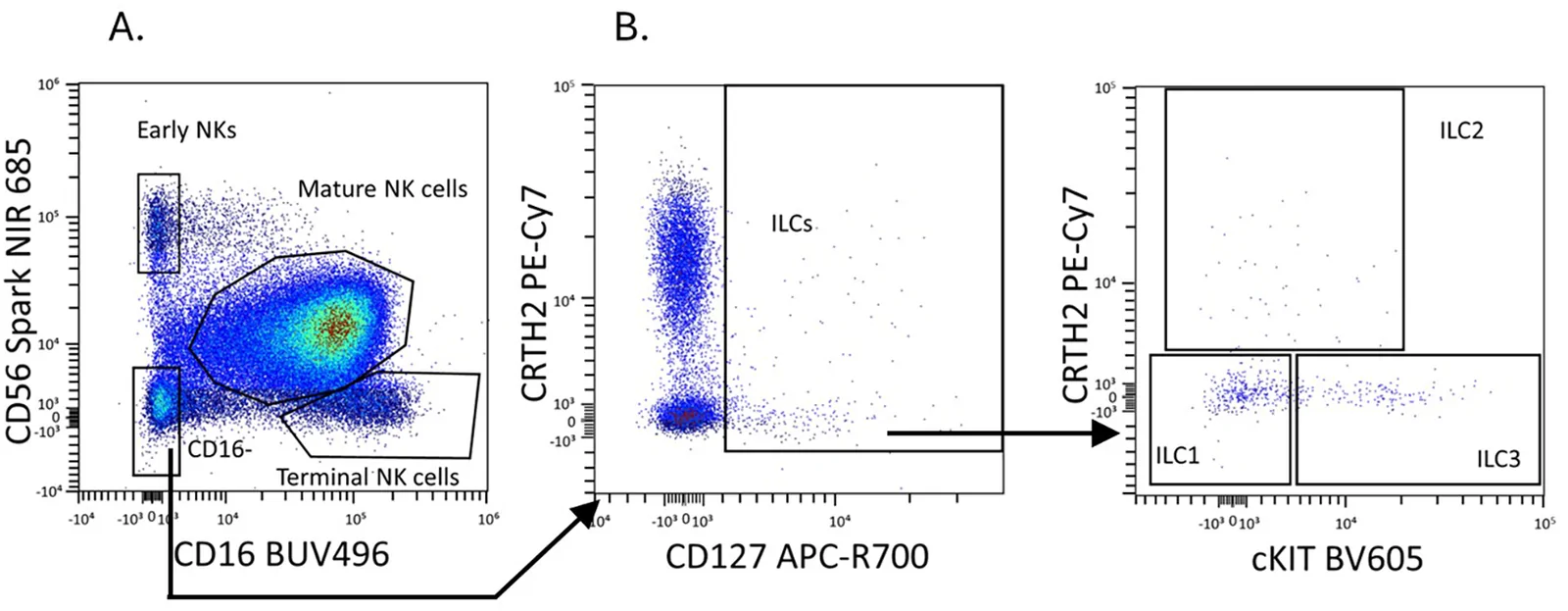
Manual gating of Natural Killer (NK) cells **(A)** and Innate Lymphoid cells (ILCs) **(B)** from viable, CD45^+^lineage^-^CD3^-^ cells.

For panel 1, viable CD45^+^lineage^-^CD3^-^ cells (**Figure 2A**) were separated using CD56 and CD16 into early (CD56+CD16-), mature (CD56+CD16+) and terminal (CD56-CD16+) NK cells (**Figure 3A**). Lineage^-^CD3^-^ CD16^-^CD127^+^ cells were identified as ILCs, which were further separated by CRTH2 and cKIT into ILC1 (CRTH2^-^cKIT^-^), ILC2 (CRTH2^+^cKIT^-^) and ILC3 (CRTH2^-^cKIT^+^) (**Figure 3B**).

**Figure 4:**
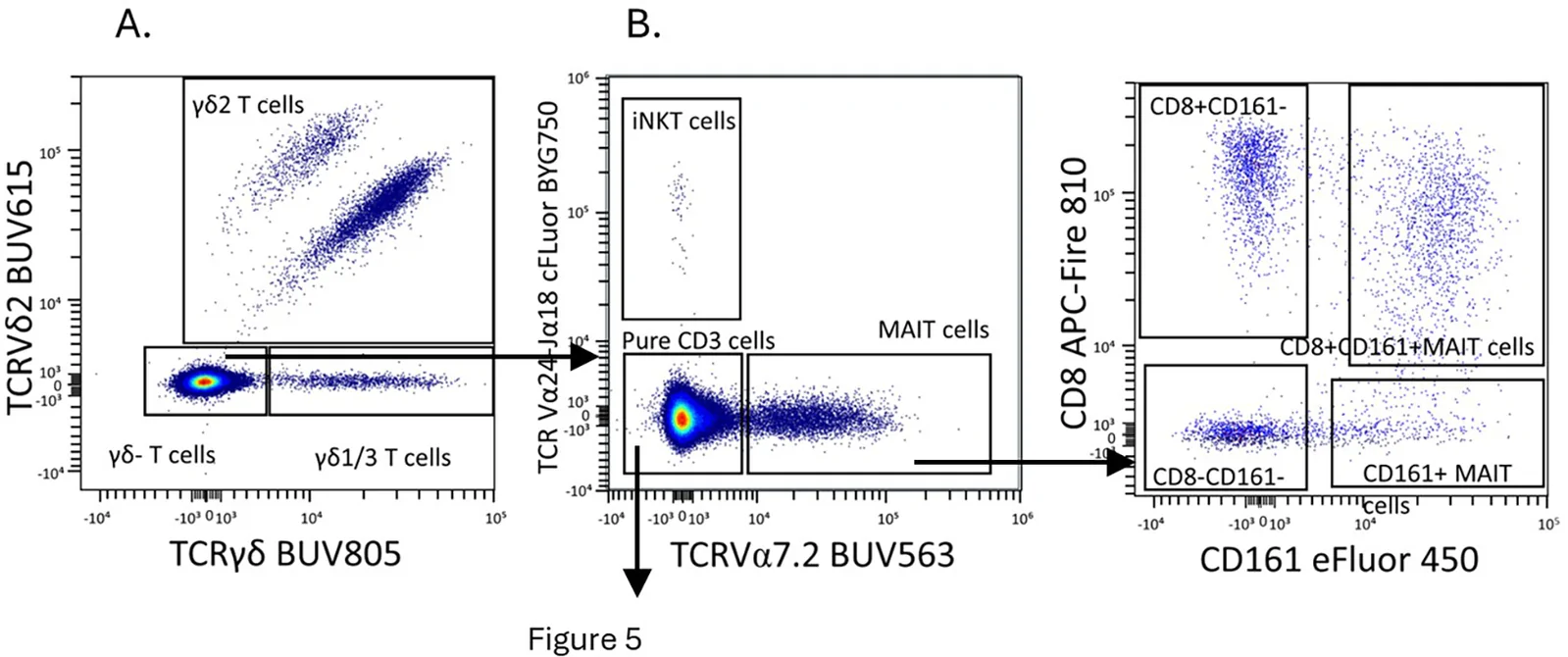
Manual gating of γδ-T cells **(A)**, innate Natural Killer T (iNKT) cells and Mucosa Associated Invariant T cells **(B)** from Viable CD45^+^ Lineage^-^ CD3^+^ cells.

The lineage^-^CD3^+^ cells (**Figure 2A**) were separated into γδ1 T cells, γδ2 T cells and γδ^-^ T cells by TCRγδ and TCRVδ2 (**Figure 4A**). From the TCRγδ^-^CD3^+^ population iNKT cells (CD3^+^TCRVα24-Jα18^+^), and CD3^+^TCRVα7.2^+^ cells were classified. TCRVα7.2^+^ T cells were further subdivided into CD161^+^ CD8^-^, and CD161^+^CD8^+^ MAIT cells and into CD161^-^CD8^-^ and CD161^-^CD8^+^ conventional cells (**Figure 4B**).

**Figure 5:**
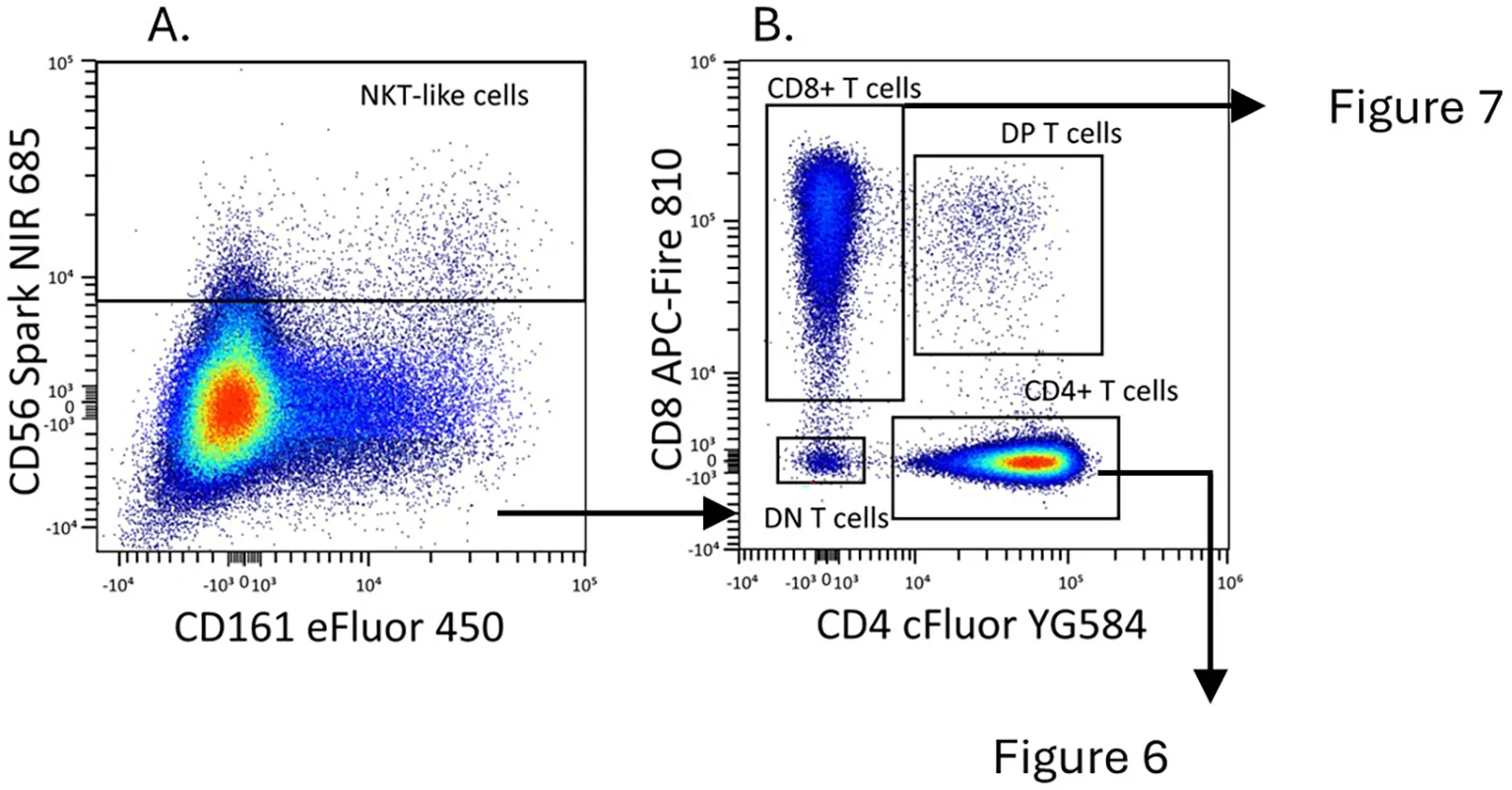
Manual gating of Natural Killer T-like (NKT-like) cells **(A)**, CD4^+^ and CD8^+^ T cells **(B)** from the CD3^+^ TCRγδ^-^ TCR Vα24-Jα18^-^ TCR TCRVα7.2^-^ population.

From the CD3^+^iNKT-TCRVα7.2^-^ cells, NKT-like cells were gated based on expression of CD56 (**Figure 5A**). Non-NKT-like cells were separated into CD8, CD4, double positive (DP) and double negative (DN) T cells (**Figure 5A**).

**Figure 6:**
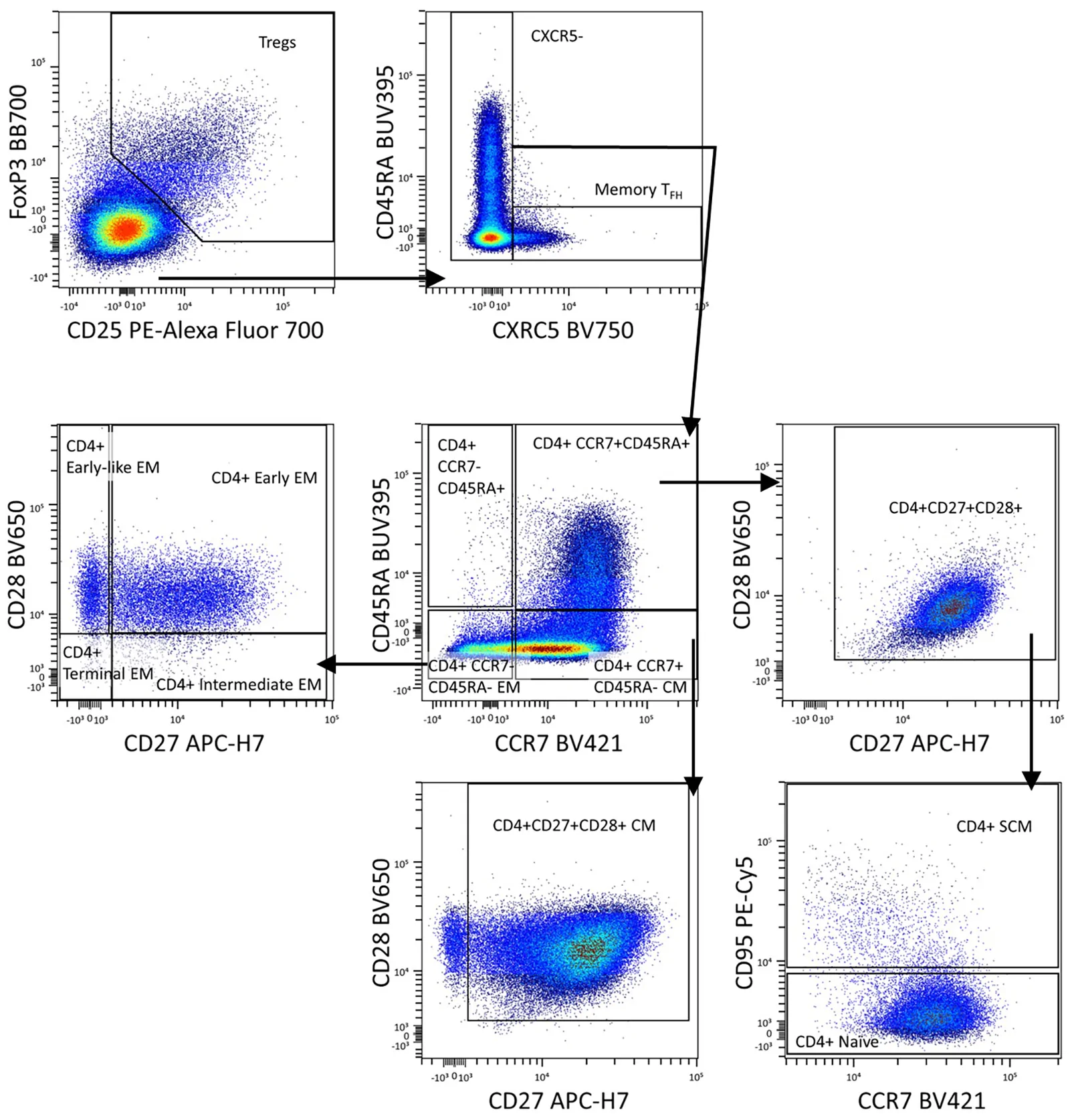
Manual gating of T regulatory cells (Tregs) and CD4^+^ naïve, memory, and effector memory (EM) subpopulations from the CD4^+^ T cells.

From the CD4^+^ T cells regulatory T (Treg) cells were identified as FoxP3^+^CD25^+^. From the remaining CD4^+^ T cells, memory T follicular helper (TFH) cells were gated as CXCR5^+^CD45RA^-^. The CD4^+^CXCR5^-^ T cells were divided by CD45RA, CCR7, CD28, CD27 and CD95 into the following populations: CD4^+^ effector memory T cells re-expressing CD45RA (TEMRA) (CD4^+^CCR7^-^CD45RA^+^), Stem cell-like memory CD4 T (Tscm) cells (CD4^+^CCR7^+^CD45RA^+^CD27^+^CD28^+^CD95^+^), Naïve CD4 T cells (CD4^+^CCR7^+^CD45RA^+^CD27^+^CD28^+^CD95^-^), central memory CD4 T (TCM) cells (CD4^+^CCR7^+^CD45RA^-^CD27^+^CD28^+^), CD4^+^ Early-like effector memory T (TEM) cells (CD4^+^CCR7^-^CD45RA^-^CD28^+^CD27^-^), CD4^+^ Early TEM cells (CD4^+^CCR7^-^CD45RA^-^CD28^+^CD27^+^), CD4^+^ Intermediate TEM cells (CD4^+^CCR7^-^CD45RA^-^CD28^-^CD27^+^) and CD4^+^ Terminal TEM cells (CD4^+^CCR7^-^CD45RA^-^CD28^-^CD27^-^) (**Figure 6**).

**Figure 7:**
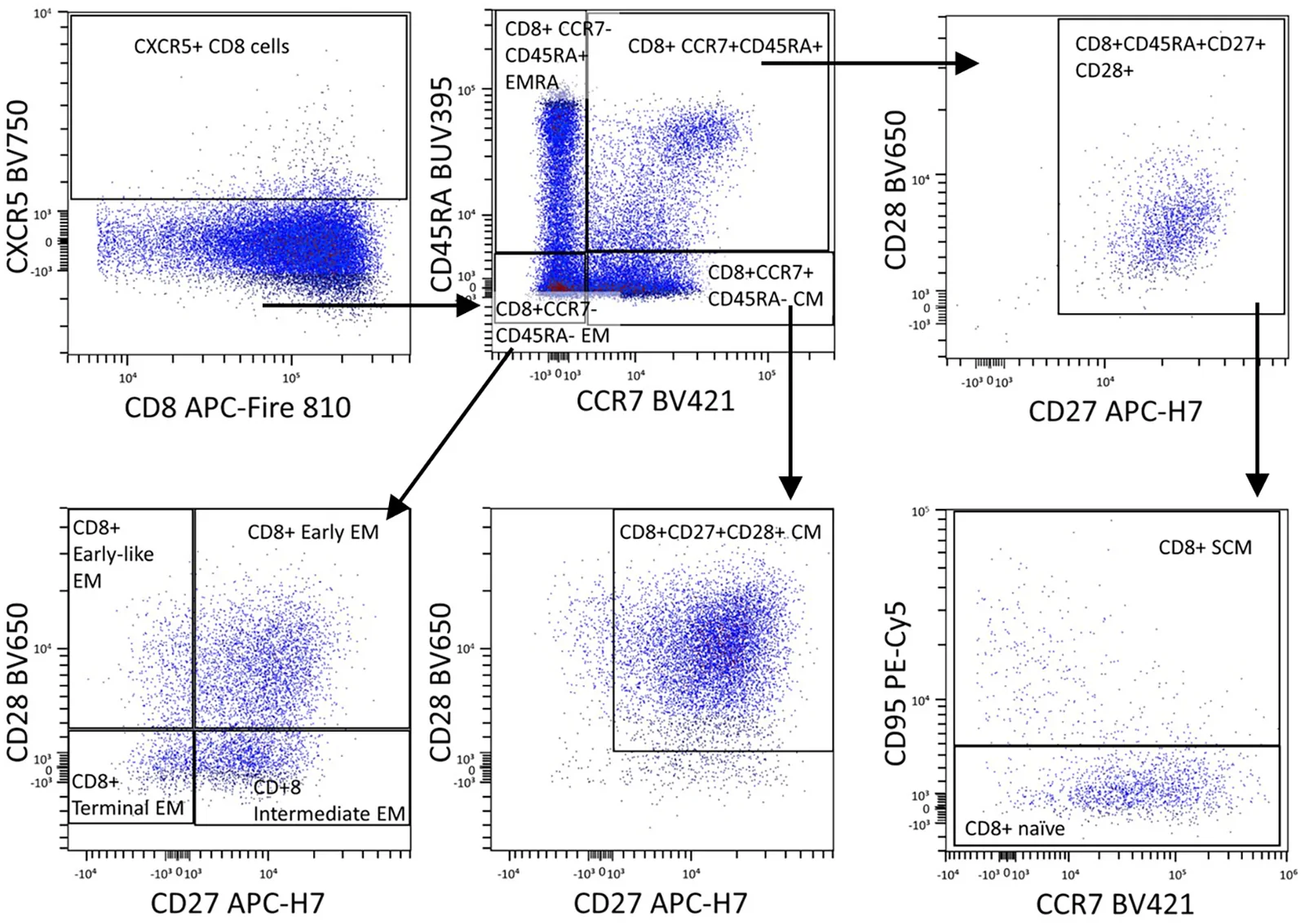
Manual gating of CXCR5^+^ CD8^+^ T cells and CD8^+^ naïve, memory, and effector memory (EM) subpopulations from the CD8^+^ T cells.

From the CD8^+^ T cells, CXCR5^+^ CD8 T cells were identified. From the CXCR5^-^ population, the following CD8 T cell subsets were resolved using CD45RA, CCR7, CD27, CD28 and CD95: CD8^+^ effector memory T cells re-expressing CD45RA (TEMRA) (CD4^+^CCR7^-^CD45RA^+^), Stem cell-like memory CD8 T (Tscm) cells (CD8^+^CCR7^+^CD45RA^+^CD27^+^CD28^+^CD95^+^), Naïve CD8 T cells (CD8^+^CCR7^+^CD45RA^+^CD27^+^CD28^+^CD95^-^), central memory CD8 T (TCM) cells (CD8^+^CCR7^+^CD45RA^-^CD27^+^CD28^+^), CD8^+^ Early-like TEM cells (CD8^+^CCR7^-^CD45RA^-^CD28^+^CD27^-^), CD8^+^ Early TEM cells (CD8^+^CCR7^-^CD45RA^-^CD28^+^CD27^+^), CD8^+^ Intermediate TEM cells (CD8^+^CCR7^-^CD45RA^-^CD28^-^CD27^+^) and CD8^+^ Terminal TEM cells (CD8^+^CCR7^-^CD45RA^-^CD28^-^CD27^-^) (**Figure 7**).

**Figure 8:**
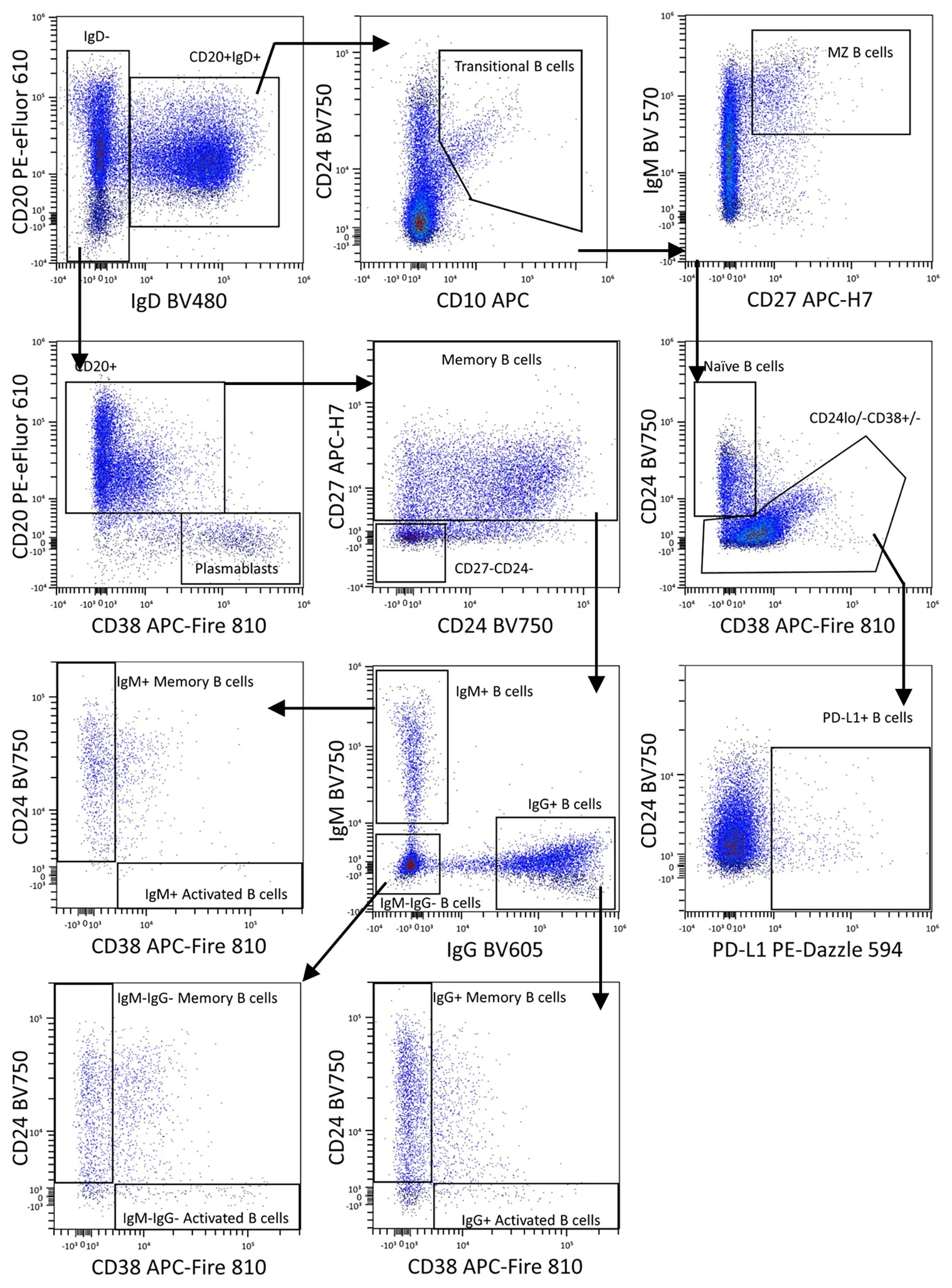
Manual gating of B cell subsets from the CD3^-^CD56^-^CD19^+^ cells.

For panel 2, Lineage^-^(CD3^-^CD56^-^CD19^-^) cells were separated from B cells (lineage^-^CD19^+^) (**Figure 2B**). B cells were further characterized by gating CD20 versus IgD. From the CD20^+^IgD^+^ cells, transitional B cells were gated based on expression of CD24 and CD10 (CD24^+^CD10^+^). In the remaining fraction of CD20^+^IgD^+^ cells, MZ B cells were gated using IgM and CD27 (IgD^+^IgM^+^CD27^+^), the rest of the cells were further divided by CD24 and CD38 expression into naïve B cells (CD19^+^CD20^+^IgD^+^CD27^-^CD24^+^) and CD34lo/-CD38^-^/^+^ cells, which are also described as regulatory B cells. PD-L1 expression was assessed on this subset. From the CD19^+^IgD^-^ cells, plasmablasts were identified using CD20 and CD38 as CD19^+^CD20^-^IgD^-^CD38^+^. From CD19^+^CD20^+^ fraction, memory B cells were gating using CD27 and CD24 (CD19^+^CD20^+^CD27^+^). Memory B cells were further subdivided into IgM^+^, IgG^+^ and IgM^-^IgG^-^ memory B cells. Using CD24 and CD38, activated (CD24^+^CD38^-^) and memory (CD24^-^CD38^+^) B cells could then be identified from the IgM^+^, IgG^+^ and IgM^-^IgG^-^ memory B cell subsets (**Figure 8**).

**Figure 9:**
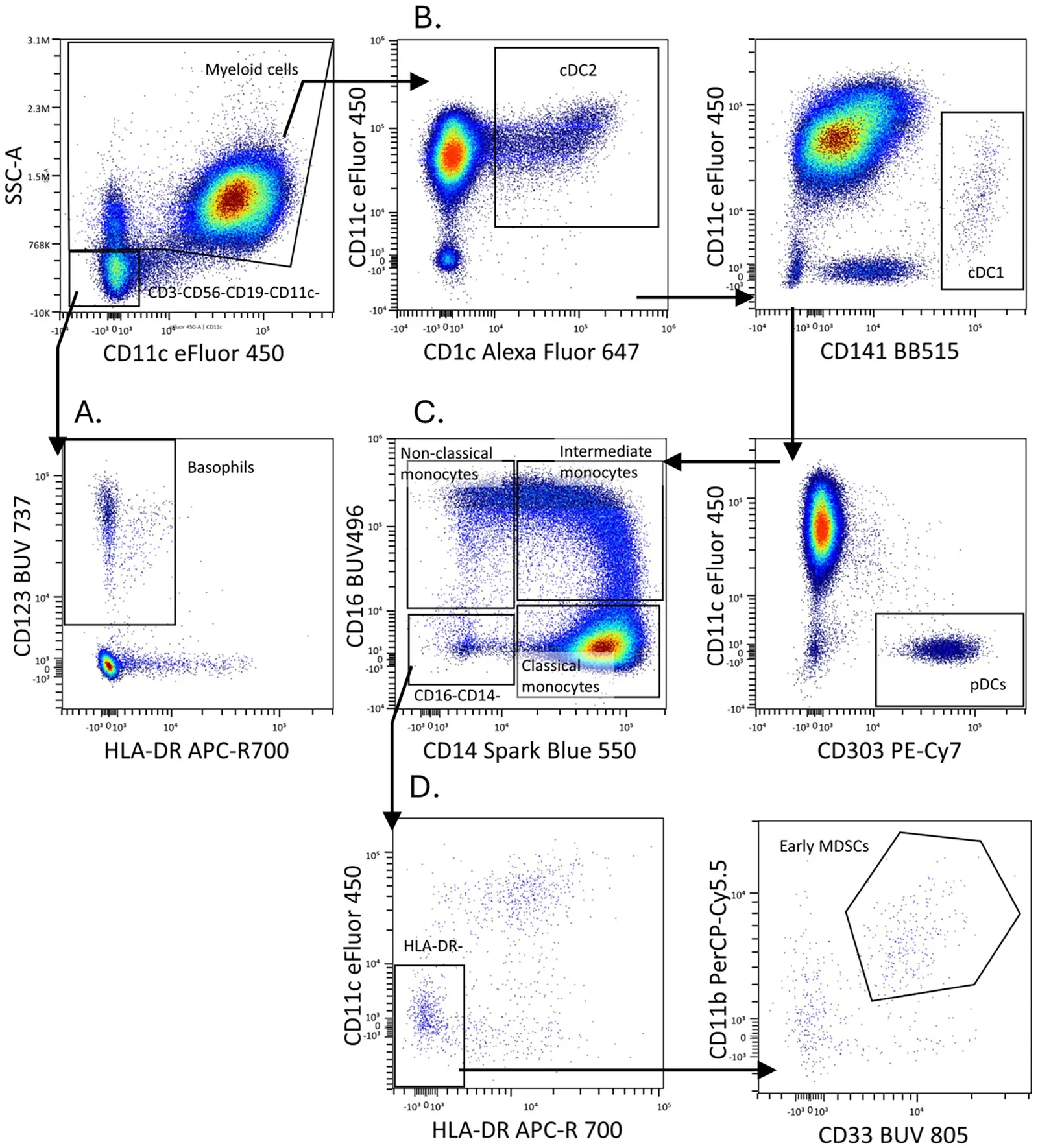
Manual gating of basophils **(A)**, Dendritic cells (DCs) **(B)**, monocytes **(C)**, and early Myeloid-derived suppressor cells **(D)** from the CD3^-^CD56^-^CD19^-^CD11c^-^ population.

Lineage^-^CD19^-^ cells (**Figure 2B**) were separated into myeloid cells and CD3^-^CD56^-^CD19^-^CD11c^-^ cells by gating based on SSC and CD11c. From the CD3^-^CD56^-^CD19^-^CD11c^-^ population, basophils were resolved (CD3^-^CD56^-^CD19^-^CD11c^-^CD123^+^) (**Figure 9A**). On the myeloid cells (4), a series of excluding gates was used to identify and gate out the following populations from the myeloid cells: CD11c and CD1c for cDC2 cells (CD11c^+^CD1c^+^), CD141 for cDC1 cells (CD1c^-^CD141^+^^+^) and CD303 for pDCs (CD1c^-^CD303^+^) (**Figure 9B**). The remaining myeloid cells were then separated using CD14 and CD16 into classical (CD14^+^CD16^-^), intermediate (CD14^+^CD16^+^) and non-classical (CD14^-^CD16^+^) monocytes and a CD16^-^CD14^-^ population (**Figure 9C**) from which early Myeloid-derived suppressor cells (MDCSs) were resolved lack of HLA-DR expression and co-expression of CD11b and CD33 (CD16^-^CD14^-^HLA-DR^-^CD11b^+^CD303^+^) (**Figure 9C**).

To evaluate inter-observer reliability of the manual gating strategy, CV% was calculated per target population across seven cytometry experts. Overall, most populations showed low CV%, indicating high agreement among observers. Populations requiring extensive hierarchical gating (e.g., ILCs) or characterized by gradual rather than discrete marker expression (e.g., terminal NK cells) showed comparatively higher CV%, reflecting reduced inter-observer agreement (**Supplementary Figure 6**). These populations require particular attention in both gating and panel design.

**Figure 10:**
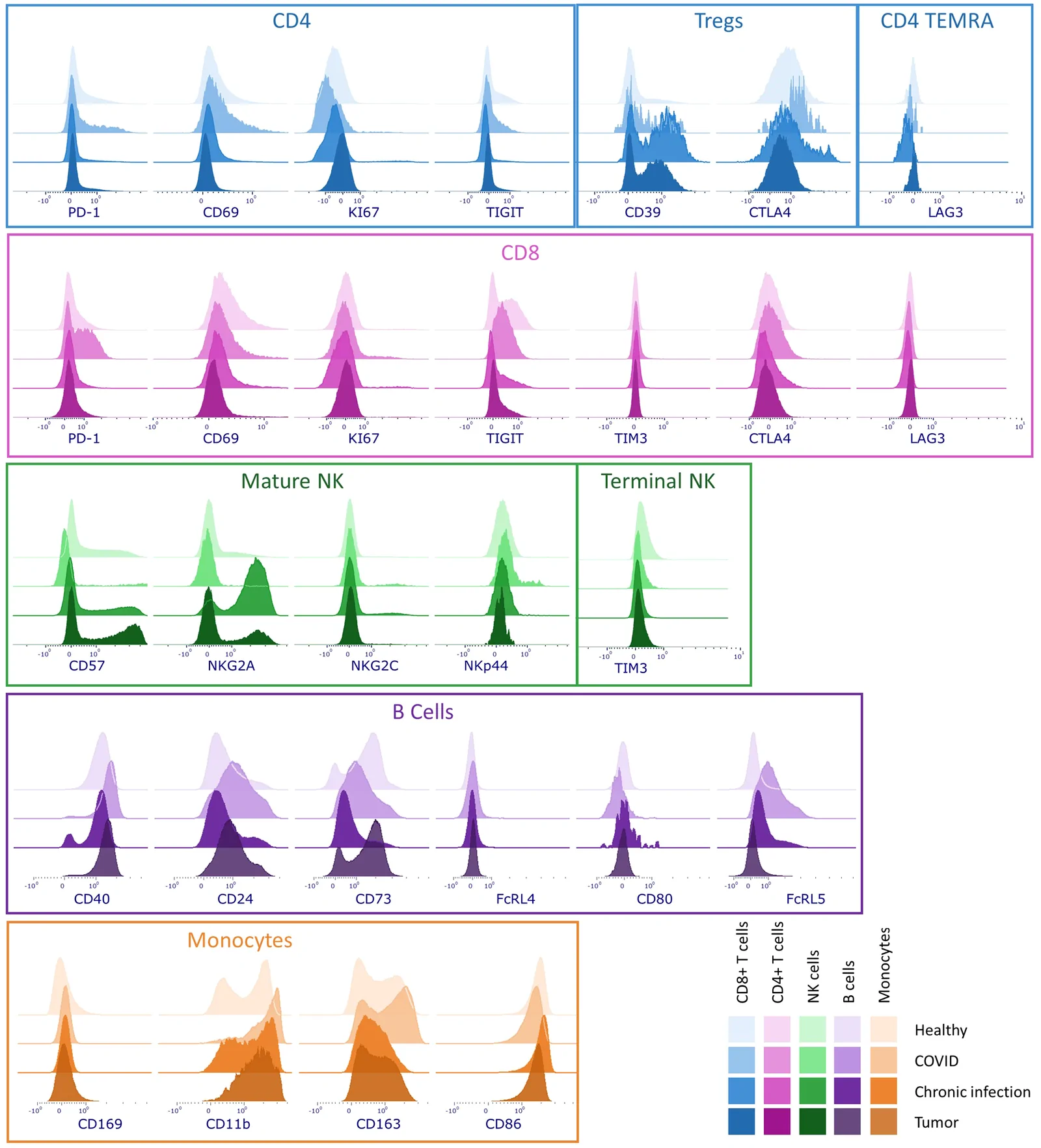
Demonstration of the panels. Staining of healthy donor (n = 8), glioblastoma (GBM) (n = 7), chronic inflammation (CI) (n = 6), and COVID-19 (n = 6) samples to show the potential of the panels. Samples were gated on CD4+ or CD8+ T cells, Regulatory T cells, CD4 terminal effector memory cells re-expressing CD45RA (TEMRA), terminal or mature NK cells, B cells or monocytes as indicated in the figure.

To demonstrate the capacity of the panels presented in this study, we stained nineteen patient samples and eight healthy donor samples to include a broad range of pathological conditions that affect immune cell composition and functional status. The expression levels of several immune checkpoints on CD4+ and CD8+ T cells and activation markers on NK cells, B cells and monocytes are shown in **Figure 10**. The altered expression of immune checkpoint markers in the presented samples from Corona virus disease (COVID) and chronic inflammation (IBD) patients is congruent with previously published results describing increased expression of immune checkpoints in patients suffering from various infections such as SARS-CoV-2 and chronic hepatitis (92–95).

### 4.3 Unsupervised analysis

**Figure 11:**
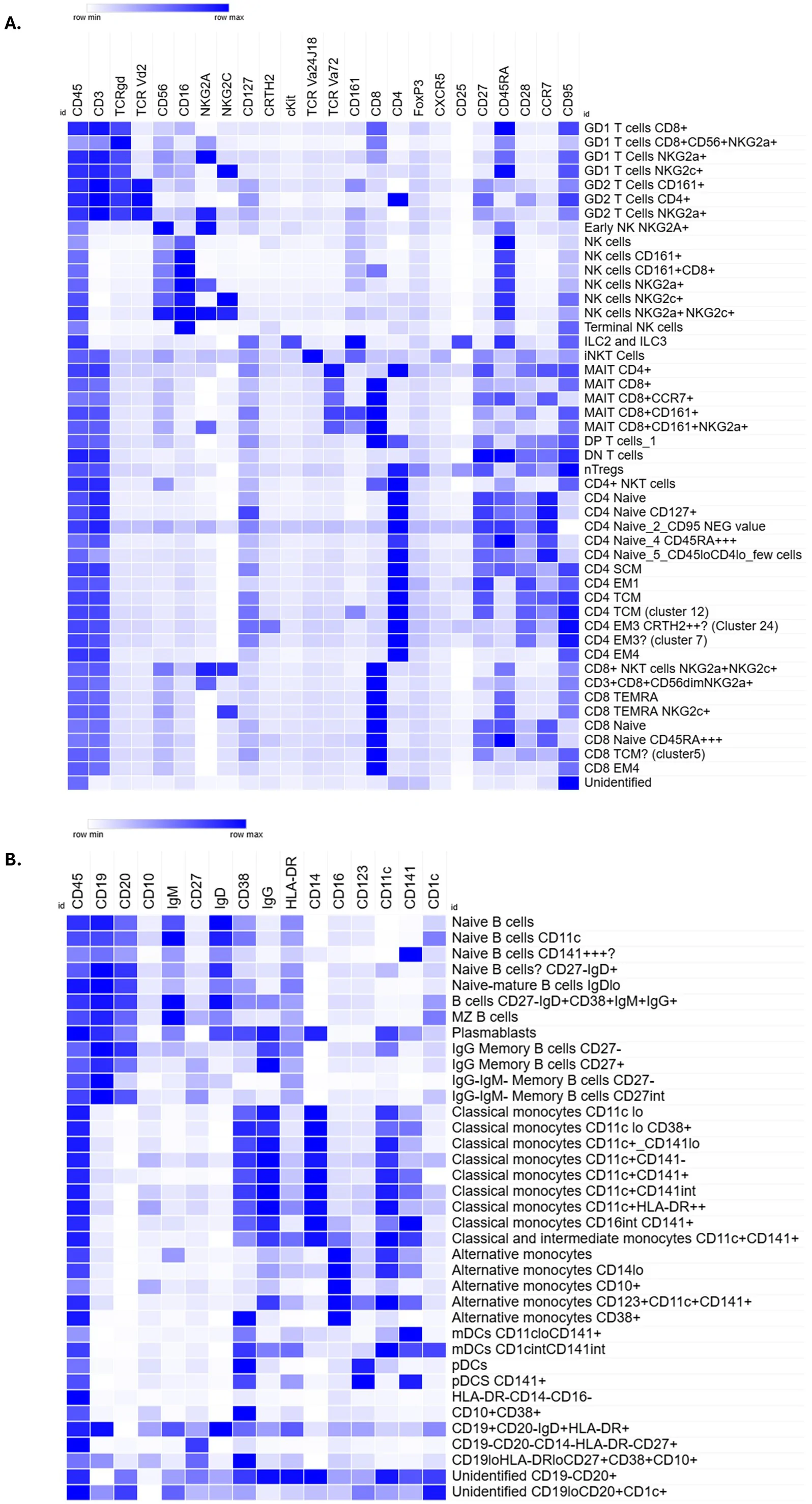
Clustered heatmaps. Clustered heatmaps showing the Median Fluorescence Insensities (MFIs) of lineage markers applied for UMAP generation and Phenograph clustering used for manual allocation of Phenograph clusters from 33 (P1) and 32 (P2) concatenated samples for panel 1 **(A)** and 2 **(B)**. Expression intensity is displayed on a colour scale from white (negative) to blue (positive).

**Figure 12:**
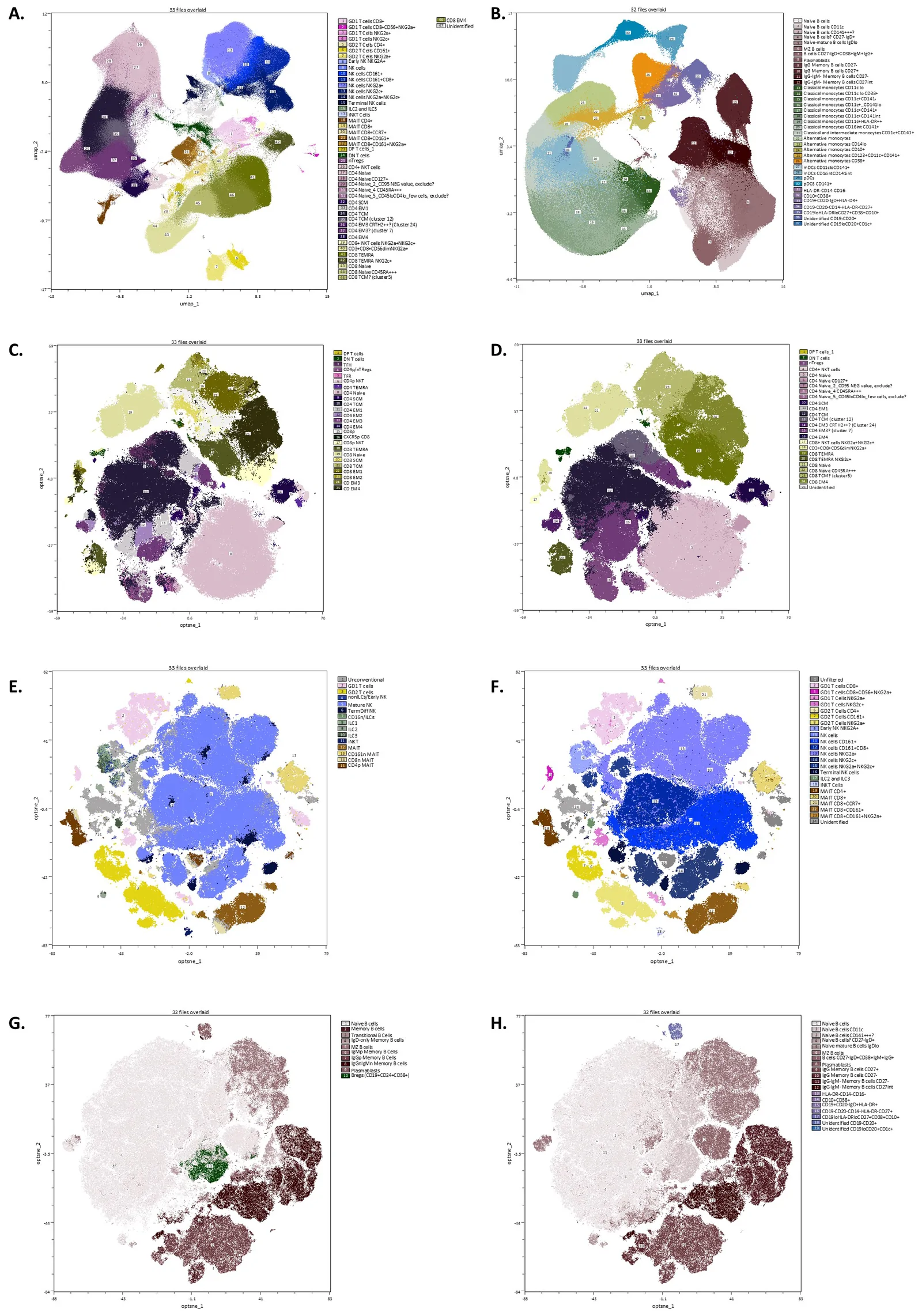
Phenograph clusters projected onto a Uniform Manifold Approximation and Projection (UMAP) plot with manual annotation of the clusters based on heatmap analysis for panel 1 **(A)** and 2 **(B)**. Additional unsupervised analysis showing optimized t-Distributed Stochastic Neighbor Embedding (opt-SNE) plots of conventional T cells **(C, D)**, unconventional T cells **(E, F)**, and B cells **(G, H)** comparing manual gating annotation [left, **(C, E, G)**] to annotated Phenograph clusters (right, **(D, F, H)**.

In addition to the manual gating, unsupervised analysis was applied for an additional unbiased and in-depth immune profiling on all included healthy donor and patient samples. UMAP (90) was applied for dimensionality reduction, followed by Phenograph clustering (96). Phenograph clusters were annotated using clustered heatmap analysis, using a heatmap projecting the normalized MFIs of the lineage markers in each cluster (**Figure 11**) and overlayed onto the generated UMAP (**Figures 12A, B**). This unsupervised analysis provided additional granularity compared to the manual gating strategy, particularly for NK cells, γδ T cells and MAIT cells, allowing for more detailed phenotyping of these subsets. For the CD8 and CD4 T cells, the resolution for some of the populations was lost however, specifically for the EM subtypes.

To further investigate the performance of the unsupervised analysis at the lineage level, additional clustering using opt-SNE was applied to subsets of the data, namely T cells, unconventional T cells, and B cells. The resulting opt-SNE projections were overlayed with both the original Phenograph cluster allocations and the manual gating annotations, allowing direct comparison between the two approaches (**Figures 12C–H**). Applying opt-SNE at the lineage-subset level provided increased granularity compared to the whole-dataset unsupervised analysis, enabling more in-depth characterization of immune cell populations within each lineage, with high agreement to the manual gating strategy.

**Figure 13:**
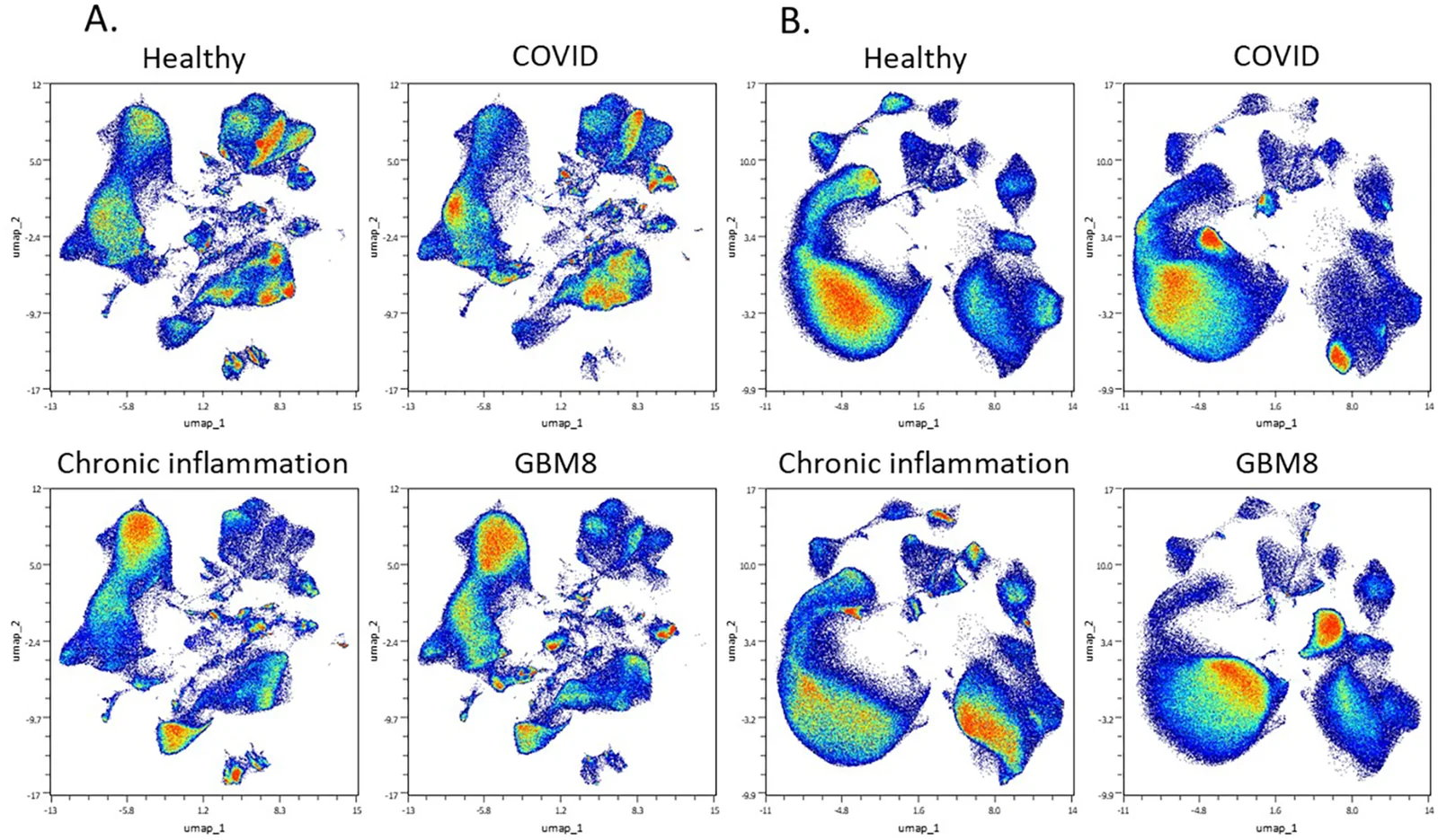
Uniform Manifold Approximation and Projection (UMAP) projection of T cells, Natural Killer (NK) cells, Innate Lymphoid Cell (ILCs), Mucosal-associated invariant T (MAIT) cells **(A)**, B cells, Dendritic Cells (DCs) and monocytes **(B)** for all samples concatenated per condition and overlaid.

To showcase the potential of the current panels, changes in immune cell populations between disease conditions and healthy controls were visualized by showing populations of the concatenated files of each condition on the generated UMAP for both of the panels. Combining data for all patients revealed distinct patterns in γδ T cells between patient samples and healthy controls and disease (**Figure 13A**), of classical and alternative monocytes in COVID-19 patients compared with healthy donors and other disease conditions and of IgG^-^IgM^-^ Memory B cells in GBM patients as compared to healthy controls, COVID-19 patients and chronic inflammation patients. This shows that our panels are capable of capturing differential immune profiles for disease and healthy conditions.

## 5 Discussion

In this study, we present a comprehensive immunophenotyping method based on two complementary spectral flow cytometry panels encompassing 60 unique markers for the in-depth characterization of T cells, γδ T cells, NK cells, MAIT cells, ILCs, B cells, monocytes, and dendritic cells, including exhaustion, differentiation and activation status. This strategy enables extended immune profiling of PBMCs that could be applied to immune monitoring studies aiming to identify cellular diagnostic or prognostic biomarkers in different patient cohorts or to understand the efficacy of immunotherapy in the treatment of tumors or chronic inflammatory or autoimmune diseases.

The method was designed for use on the Cytek Aurora 5-laser instrument, taking into account biological relevance and optimal resolution of the selected markers, while minimizing spectral overlap. The current method was developed and evaluated in accordance with previously published strategies, using well defined metrics for panel design (5, 79, 97). Together, the panels enable the resolution of more than 50 phenotypically distinct immune populations within human PBMCs, including rare subsets and populations requiring extensive marker combinations, as well as the assessment of differentiation, activation, and exhaustion states.

Dividing the markers across two complementary panels offers several advantages over a single large panel. It allows for inclusion of a greater total number of markers while maintaining marker resolution and minimizing problematic spillover spread. This is especially relevant for rare subsets which need extensive marker combinations to properly identify them. ILCs for example, are key regulators of mucosal immunity and tissue homeostasis, and dysregulation of these cells is linked to pathogenesis of various cancers and auto-immune diseases. This makes them essential cells to study, however, to resolve ILC, the most abundant circulating subsets have to be excluded first (43).

We applied both manual gating and unsupervised analysis, which allowed for cross-validation of the manual gating strategy and provided additional, unbiased insight into the immune cell populations present in the samples. Manual gating remains the leading force in panel design, as it enables direct, hypothesis-driven validation that a panel can effectively resolve the desired target populations. Unsupervised clustering, in turn, facilitates discovery and accelerates analysis, but is most informative once a panel has been properly validated through manual gating. Each approach offers distinct advantages. Manual gating allows for detailed characterization of activation states within individual, well-defined subsets, but that are unlikely to be captured through unsupervised analysis alone. Unsupervised clustering, conversely, can reveal additional heterogeneity within subsets that would be difficult to delineate through conventional gating. The unsupervised analysis was able to resolve additional phenotypic granularity within several lineages, most notably NK cells, γδ T cells and MAIT cells. This suggests that these populations may in fact comprise several biologically distinct subsets, and further characterization of these clusters is warranted to determine their biological relevance. For other lineages, such as CD8 and CD4 T cells, resolution of specific subsets (e.g., EM subtypes) was reduced compared to manual gating. To further resolve this at the lineage level, opt-SNE was applied to subsets of the data (T cells, unconventional T cells, and B cells), which improved granularity and yielded good agreement with the manual gating annotations within these lineages. The panels were applied to healthy donor and patient samples, showing distinct immune cell profiles for the patient samples in compared to the healthy controls. Although the samples were acquired over different batches, no problematic differences were observed in between samples from different batches after batch correction, underlining the reproducibility of our method. This is especially relevant for multicenter, large and/or longitudinal studies where samples are collected and processed at separate timepoints or in different batches.

A key limitation of the current method is the long staining protocol, which is inevitable for a complementary method consisting of two panels including both surface and intracellular staining steps. Both panels require sequential staining steps, which contribute to the length of the staining protocol. However, the staining protocol can be divided over two days, once the fixation/permeabilization is concluded, intracellular staining can be done the next day, significantly reducing the time needed within one day.

Contributing to the length of the staining protocols, is the sequential staining included for TCRs, cytokines and IgG. Losses of resolution related to the order in which antibodies are added, are a challenge in designing high dimensional flow cytometry panels as a result of steric hindrance, non-specific binding and interference as discussed before (5, 25, 98, 99). In accordance with these previously published studies, our work highlights the importance of considering sequential staining as a tool to improve marker resolution in complex panels such as presented here.

Another limitation of this protocol is that it was validated only on PBMCs. For application of the protocol to other sample types, marker inclusion has to be adjusted to include relevant markers for the sample type of interest. However, when marker selection is adjusted, fluorochrome assignment, antibody titrations and panel optimization also have to be re-evaluated. Furthermore, a minimum cell input of 1x10^6^ viable PBMCs is regarded essential for application of both panels. This limit was selected as we aim to resolve rare subsets, populations with a frequency of ≤0.01% (100, 101), such as iNKT cells, ranging in frequency from 0.01–1% of PBMCs (102–106). No strict consensus exists on the limit of detection for analysis of cell populations with low frequencies (107–109), but we selected the cut-off of 5x10^5^ cells per panel for the analysis of rare events. This minimum cell input of 1x10^6^ viable PBMCs may limit applicability in studies with very restricted sample volumes. In such cases, a single panel can be used, reducing the minimum input to 5x10^5^ cells.

The current method was designed for the Cytek Aurora 5-laser instrument. When Cytek Assay Settings (CAS) are used, the method should be reproducible on other Cytek Aurora 5-laser instruments. It is recommended however to run RCs on each instrument that is used for sample acquisition and to use those RCs specifically for unmixing. However, the panels were not tested on comparable instruments from other manufacturers. Although comparable results between different platforms have been reported (110), additional panel optimization and adaptations to the panels may be necessary to adapt the panels for use on another instrument type to obtain similar results as presented here. As instruments continue to improve, the value of this combined strategy remains, because the improvement in technology will result in lower complexity of the panels. This translates to higher resolution. For that reason, the current approach is likely to remain useful for a long time. Additionally, it allows for adding additional markers as instruments improve, possibly with deep UV and infra-red dyes.

In summary, we have designed comprehensive spectral flow cytometry approach that can be used for immune profiling of PBMCs in immune monitoring studies aiming to identify cellular diagnostic or prognostic biomarkers. In addition, we provide detailed information on panel design, evaluation of marker resolution, manual gating and options for unsupervised analysis, which can be used to adapt the panels to the user’s requirements.

## Data Availability

The datasets presented in this study can be found in online repositories. The names of the repository/repositories and accession number(s) can be found below: 10.5281/zenodo.19796694.
