# Supplementary material for: Comprehensive immune profiling of human peripheral blood mononuclear cells using two complementary spectral flow cytometry panels encompassing 60 unique markers
Source: Front Immunol. 2026 Sep 1;17:1894779. doi: 10.3389/fimmu.2026.1894779 (PMC13574664; doi:10.3389/fimmu.2026.1894779)
Supplement: Supplementary file 1 [file Image2.pdf]

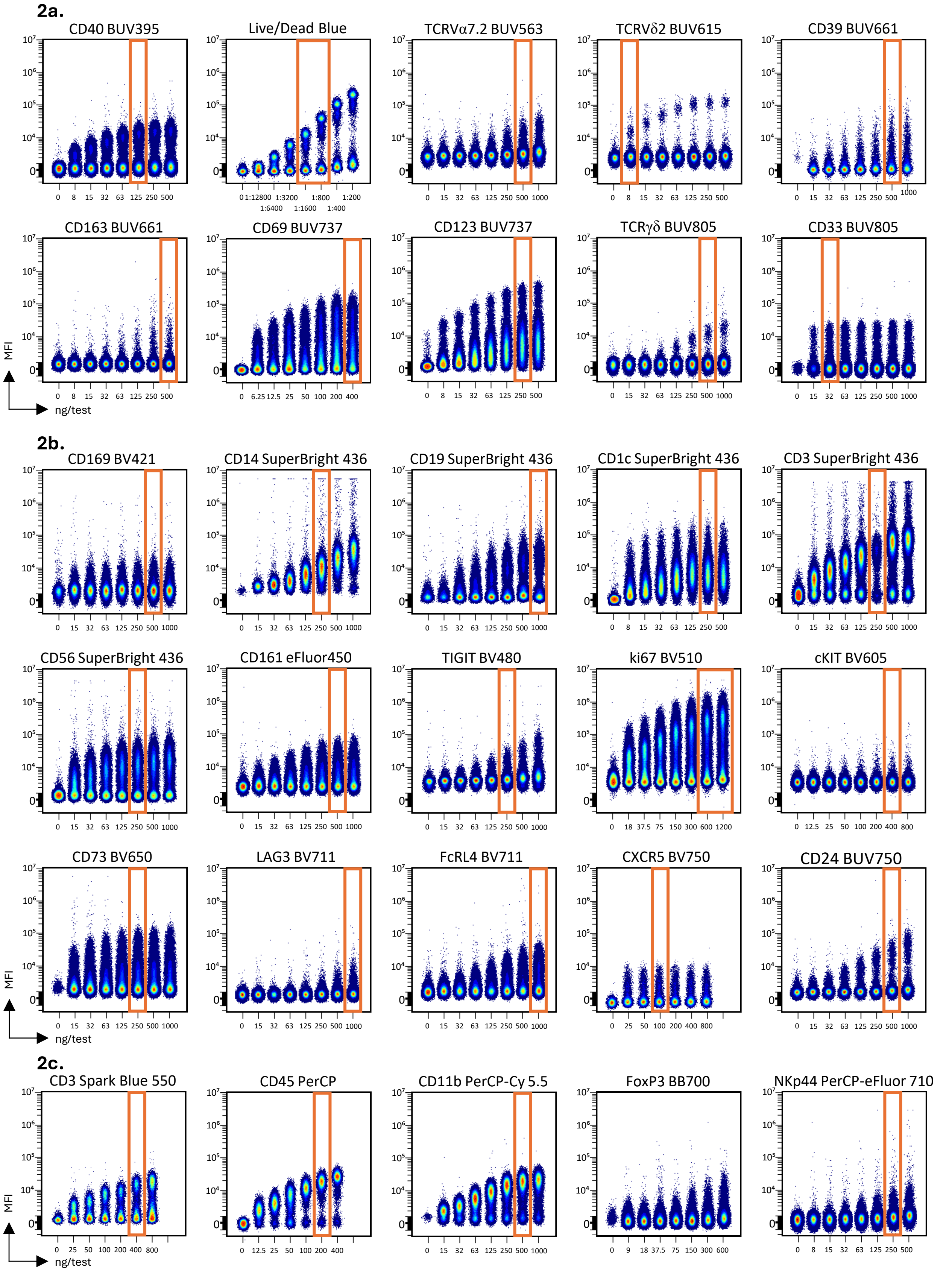
2a.
CD40 BUV395
Live/Dead Blue
TCRVα7.2 BUV563
TCRVδ2 BUV615
CD39 BUV661
CD163 BUV661
CD69 BUV737
CD123 BUV737
TCRγδ BUV805
CD33 BUV805
MFI
ng/test
2b.
CD169 BV421
CD14 SuperBright 436
CD19 SuperBright 436
CD1c SuperBright 436
CD3 SuperBright 436
CD56 SuperBright 436
CD161 eFluor450
TIGIT BV480
ki67 BV510
cKIT BV605
CD73 BV650
LAG3 BV711
FcRL4 BV711
CXCR5 BV750
CD24 BUV750
MFI
ng/test
2c.
CD3 Spark Blue 550
CD45 PerCP
CD11b PerCP-Cy 5.5
FoxP3 BB700
NKp44 PerCP-eFluor 710
MFI
ng/test

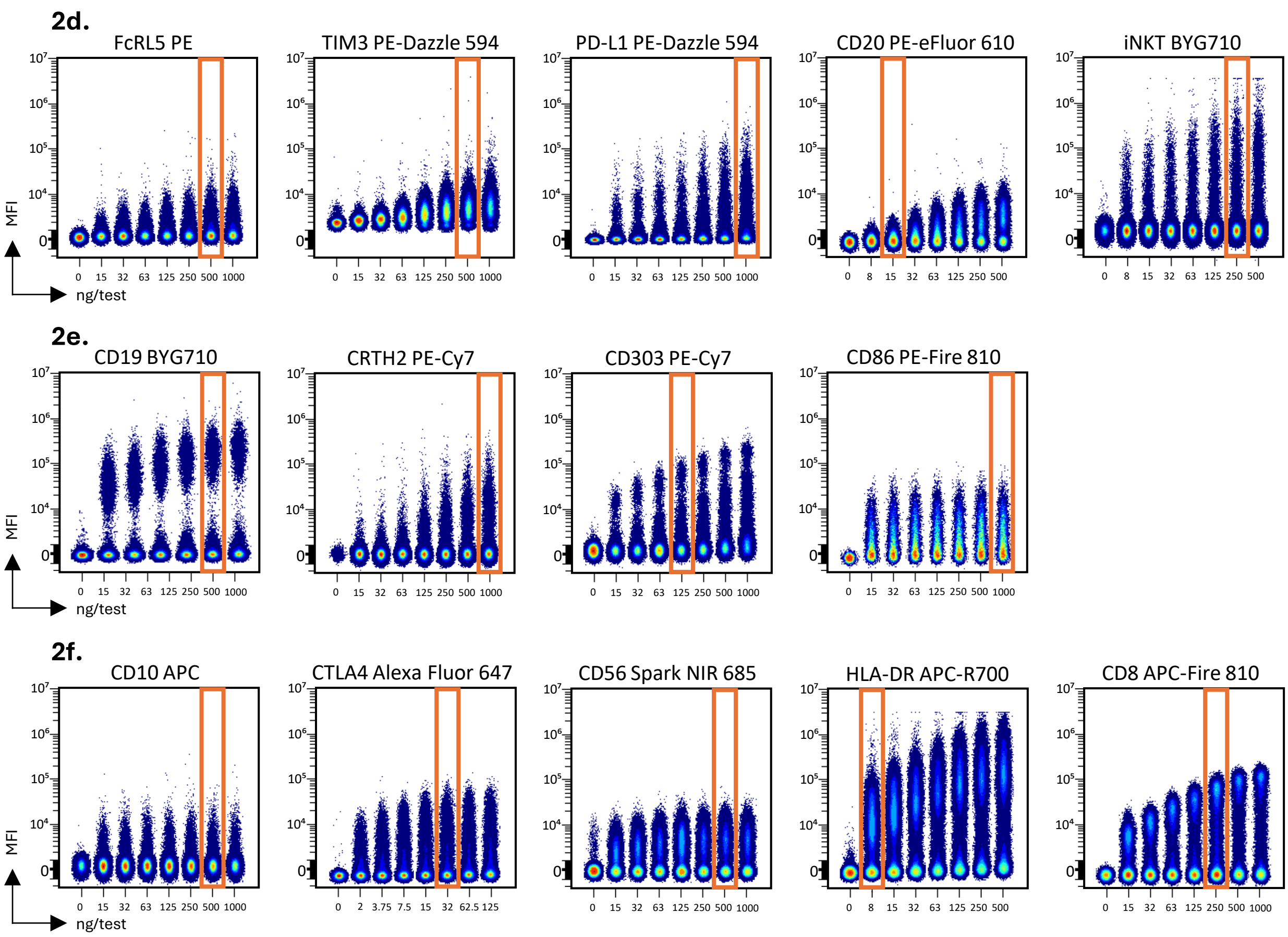

Supplementary Figure 2. Antibody Titrations. *Results of titration experiments preformed during panel development and optimization to determine optimal antibody titration for each reagent in the panels. All antibodies were tested in 2-fold dilutions ranging from 15 ng/test to 1000 ng/test or from 8 ng/test to 500 ng/test. FSC files were concatenated in OMIQ (Dotmatics). Median Fluorescence Intensity (MFI) is shown for each antibody concentration tested in ng/test. Antibody titrations are displayed corresponding to their primary exciting laser: UV laser (355 nm) (A), (A), violet laser (405 nm) (B), blue laser (488 nm) (C), yellow-green laser (561 nm) (D), and red laser (640 nm) (E). The titers selected in the full panel are indicated by the orange highlights. For some antibodies two titers are highlighted, in these cases, an intermediate titer was selected (1:2000 dilution for Live/Dead Blue, 750 ng/test for ki67). For FoxP3, no titer was highlighted, in this case, a 2-fold of the highest titer was selected (1200 ng/test) after evaluation of the resolution of the marker in the full panel.*
