# Supplementary material for: Comprehensive immune profiling of human peripheral blood mononuclear cells using two complementary spectral flow cytometry panels encompassing 60 unique markers
Source: Front Immunol. 2026 Sep 1;17:1894779. doi: 10.3389/fimmu.2026.1894779 (PMC13574664; doi:10.3389/fimmu.2026.1894779)
Supplement: Supplementary file 2 [file Image3.pdf]

A.

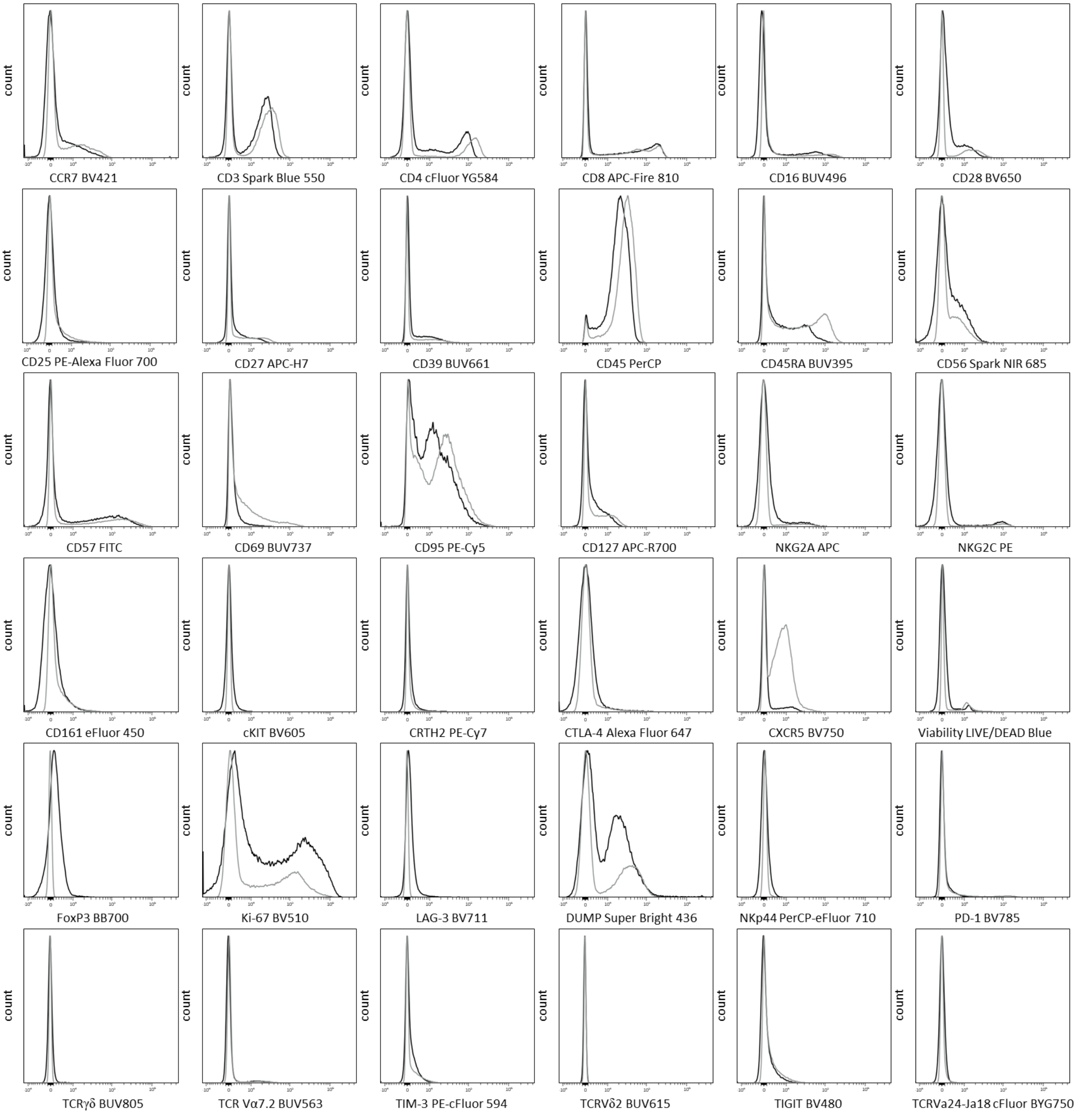
count
CCR7 BV421
CD3 Spark Blue 550
CD4 cFluor YG584
CD8 APC-Fire 810
CD16 BUV496
CD28 BV650
CD25 PE-Alexa Fluor 700
CD27 APC-H7
CD39 BUV661
CD45 PerCP
CD45RA BUV395
CD56 Spark NIR 685
CD57 FITC
CD69 BUV737
CD95 PE-Cy5
CD127 APC-R700
NKG2A APC
NKG2C PE
CD161 eFluor 450
cKIT BV605
CRTH2 PE-Cy7
CTLA-4 Alexa Fluor 647
CXCR5 BV750
Viability LIVE/DEAD Blue
FoxP3 BB700
Ki-67 BV510
LAG-3 BV711
DUMP Super Bright 436
NKp44 PerCP-eFluor 710
PD-1 BV785
TCRγδ BUV805
TCR Vα7.2 BUV563
TIM-3 PE-cFluor 594
TCRVδ2 BUV615
TIGIT BV480
TCRVa24-Ja18 cFluor BYG750

B.

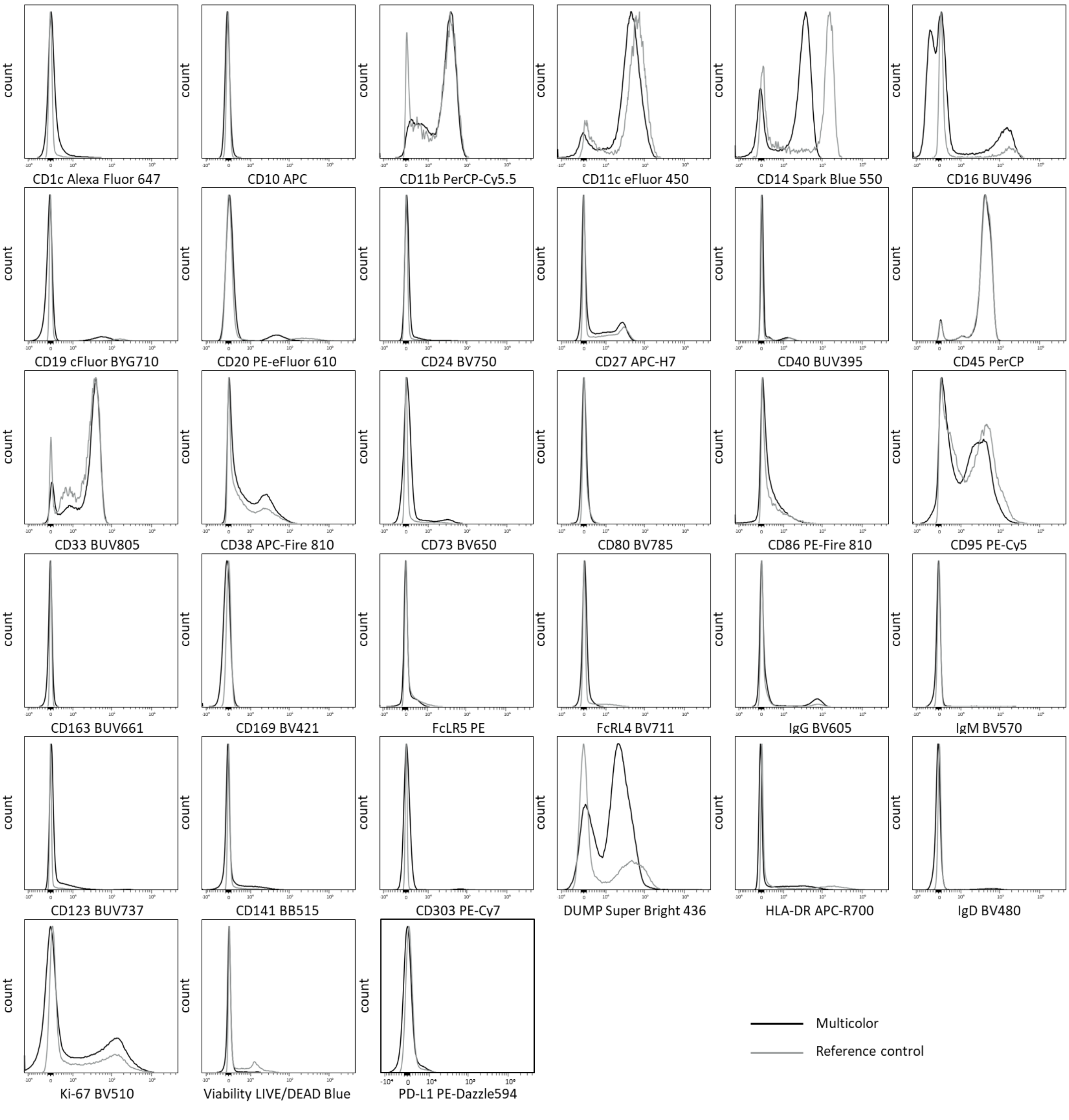

***Staining resolution Reference Control (RC) versus Multicolor (MC) samples**. To ensure optimal resolution of the markers in the MC samples, we compared the staining patterns of the unmixed RC samples (grey line) to the same marker in the unmixed MC sample (black line), all stained on PBMCs or tonsils for panel 1 (A) and 2 (B). For the markers where the positive populations very small, the histograms were not informative enough. For these markers pseudocolor plots are presented for panel 1 (C) and 2 (D). The upper plots show the RC sample, the lower plots show the MC sample. Donors for the MC sample were identical to those of the RC samples. Unmixing was done using PBMCs, tonsils or beads as noted in Online Table 6. The data shown in the overlays and pseudocolor plots are gated on single cells and, if applicable, on myeloid or lymphoid scatter gates. All plots were generated in OMIQ (Dotmatics).*
