# Supplementary material for: Comprehensive immune profiling of human peripheral blood mononuclear cells using two complementary spectral flow cytometry panels encompassing 60 unique markers
Source: Front Immunol. 2026 Sep 1;17:1894779. doi: 10.3389/fimmu.2026.1894779 (PMC13574664; doi:10.3389/fimmu.2026.1894779)
Supplement: Supplementary file 3 [file Image5.pdf]

A. Titer adjustments panel 1

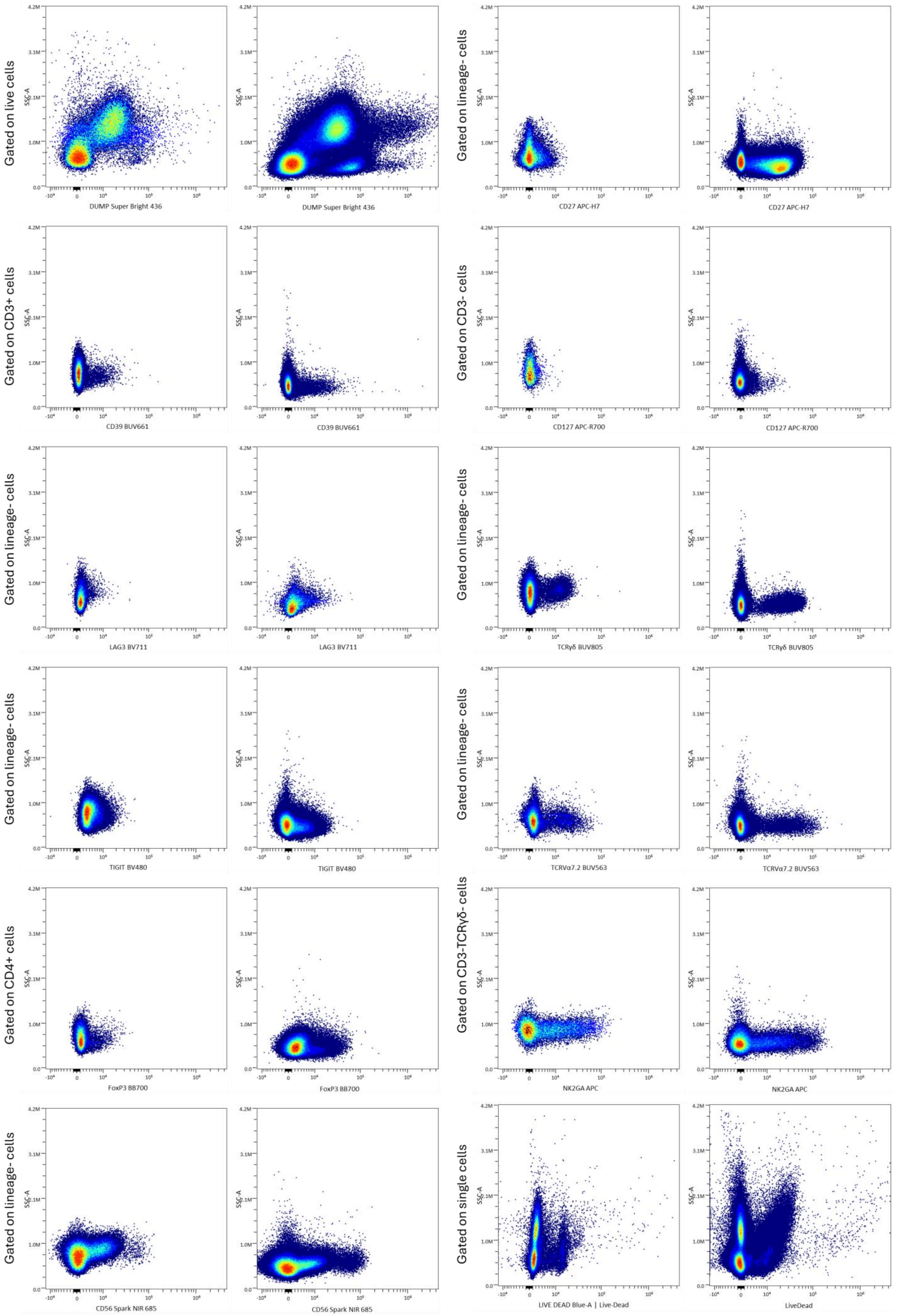

B. Loss of resolution CD161 eFluor450

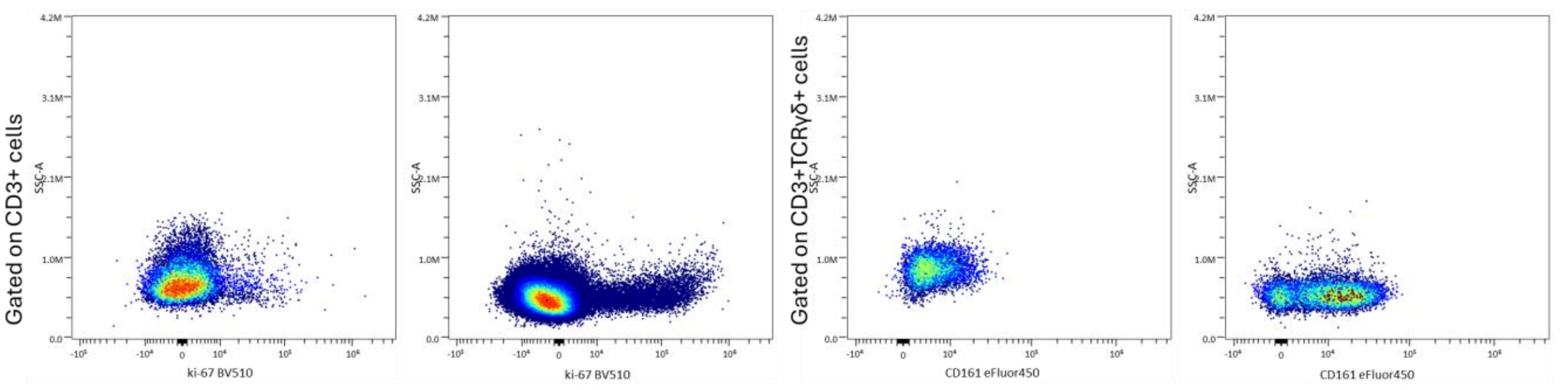

C. Titer and reagent adjustments panel 2

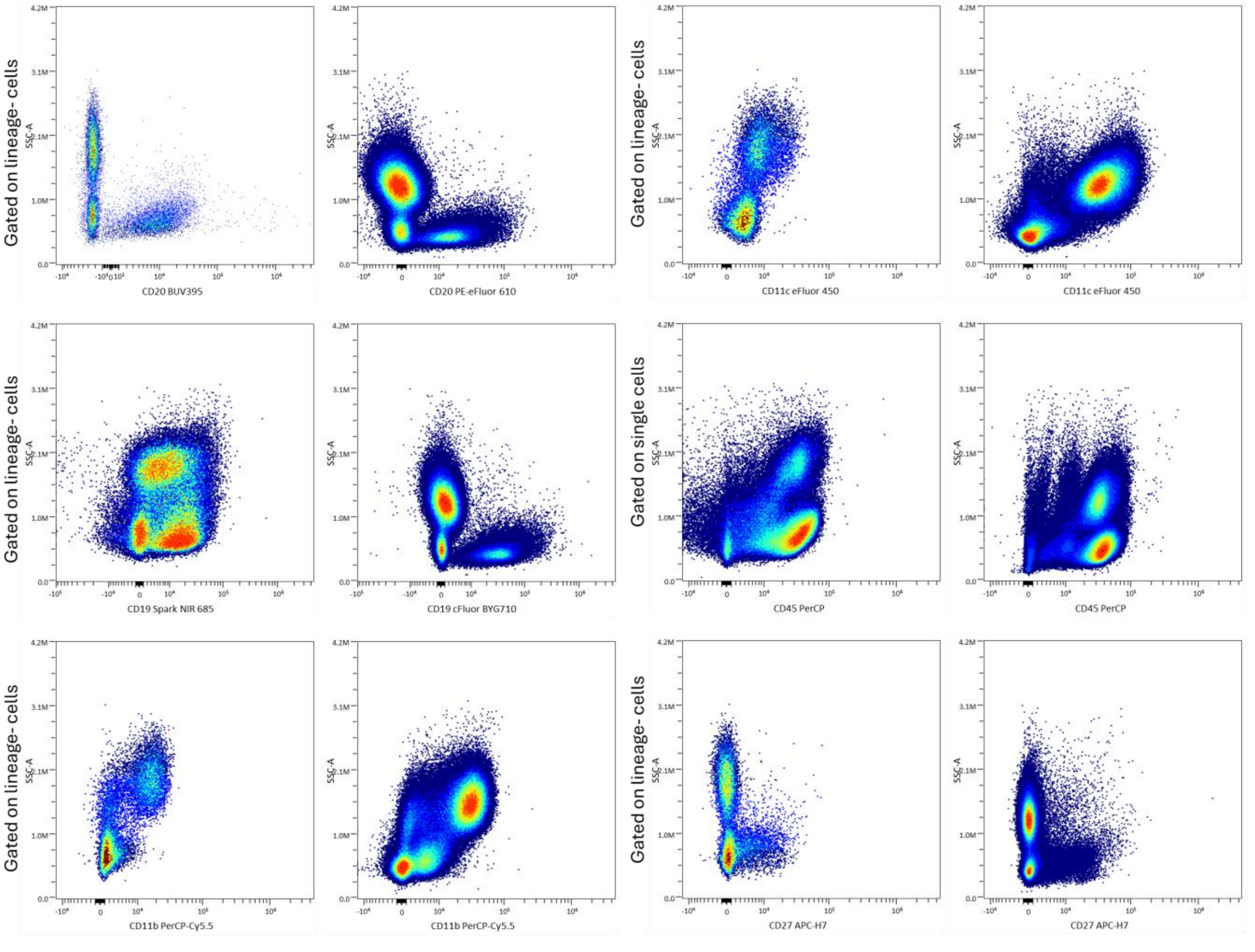

D. FcRL5 staining on monocytes

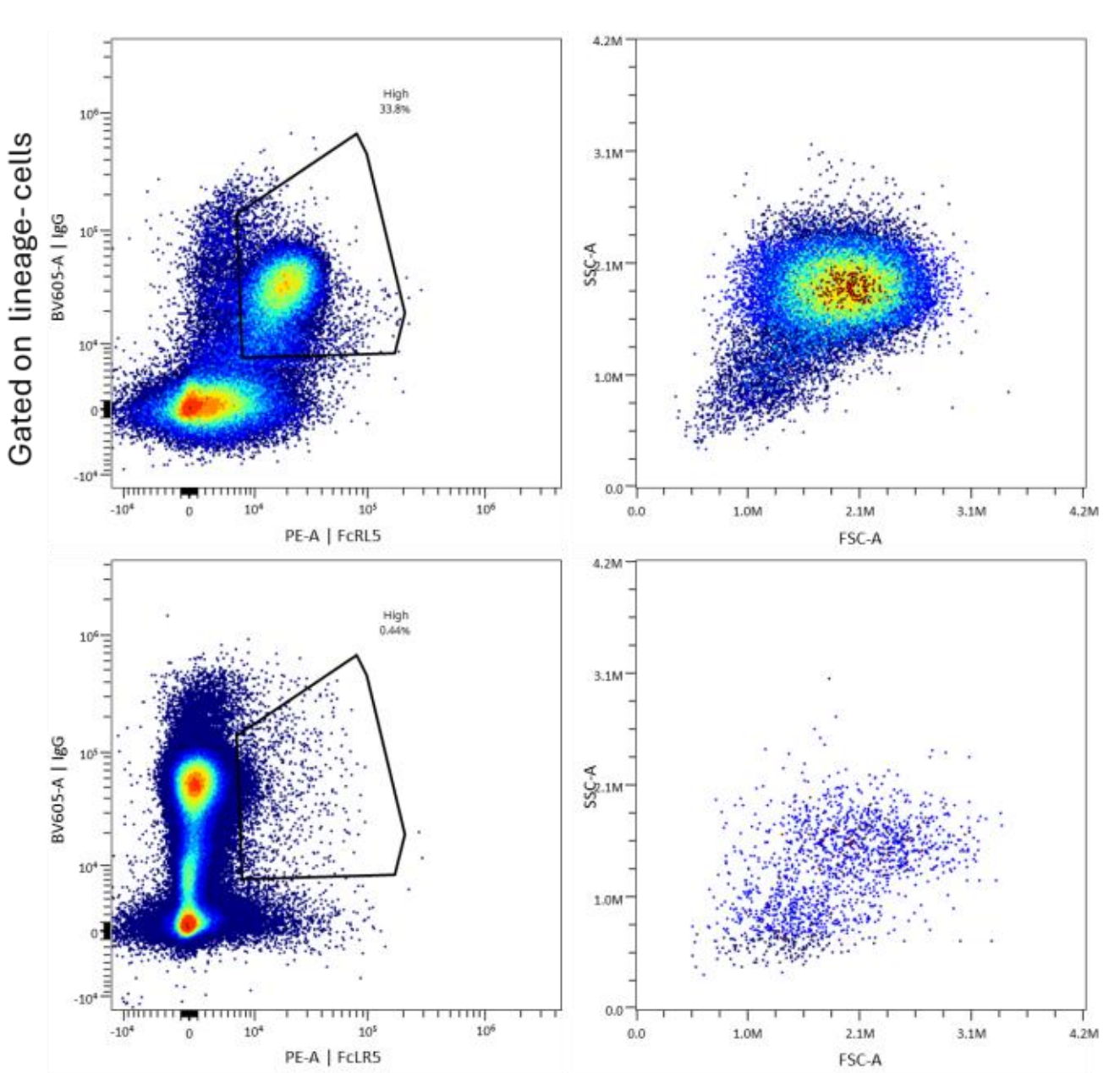

Supplementary Figure 5. Panel optimization. *Adjustments made to titers in panel 1 (A), initially selected titer is shown on the left, final titer selection is shown on the right. CD161 surface staining (B) was done simultaneously (left) and after surface staining for TCRγδ, TCRVα7.2, TCR Vα24-Jα18, CCR7 and CXCR5 (right) to test if CD161 resolution could be improved. Adjustments made to reagents and titers in panel 2 (C), initially selected reagent or titer is shown on the left, final reagent or titer selection is shown on the right. Surface staining of IgG (D) was done simultaneously with FcRL5 (upper) and prior to FcRL5 (lower), to test if the non-specific binding of IgG to monocytes resulted in the observation of FcRL5+ monocytes. Left panels shown are gated on CD56-CD3- cells. All plots are gated on PBMC samples, on the population indicated next to each set of plots. All plots were generated in OMIQ (Dotmatics).*
